# First total synthesis of caerulomycin K: a case study on selective, multiple C–H functionalizations of pyridines†

**DOI:** 10.1039/d4ra00589a

**Published:** 2024-02-13

**Authors:** Alessandro Dimasi, Mattia Failla, Arianna Montoli, Andrea Citarella, Paolo Ronchi, Daniele Passarella, Valerio Fasano

**Affiliations:** a Department of Chemistry, Università degli Studi di Milano Via Camillo Golgi, 19 20133 Milano Italy valerio.fasano@unimi.it https://www.fasanolab.com; b Medicinal Chemistry and Drug Design Technologies Department, Global Research and Preclinical Development, Chiesi Farmaceutici S.p.A Largo Francesco Belloli 11/a 43126 Parma Italy

## Abstract

Caerulomycins, natural alkaloids with antimicrobial properties, have been previously synthesized starting with highly pre-functionalized building blocks or requiring many functional group manipulations. In this work, we report the first total synthesis of caerulomycin K, a diversely trifunctionalized pyridine readily assembled in three steps exploiting the recent advancements in the C–H activation of N-heterocycles.

## Abstract 2

A radical change: the first 3-steps total synthesis of caerulomycin K was achieved exploiting Minisci chemistry using cheap starting materials.

## Graphical abstract

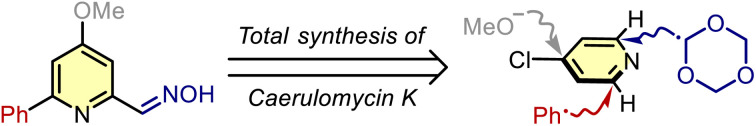

Pyridines are ubiquitous in many natural products and drugs, often with a wide selection of functionalities decorating these aromatic rings.^1,2^ While classical pyridine syntheses (*e.g.* Bohlmann–Rahtz reaction, Hantzsch condensation, *etc.*) allow the introduction of substituents in the final ring, the functionalization of existing pyridines using C–H activation is usually a better option to avoid the *de novo* synthesis of complex pyridines.^3^ However, despite pyridines exhibiting a clear similarity to benzenes, they present distinct challenges when it comes to their C–H functionalization.^4,5^ As a result, relatively simple pyridines may require several steps to be synthesized, especially if the substituents around the aromatic ring are different in nature. This is the case for caerulomycins (and related collismycins), a class of natural alkaloids produced by *Streptomyces caeruleus* and endowed with antimicrobial properties (Scheme 1A).^6–11^ For instance, taking caerulomycin E as the prototype of this type of bioactive compounds, different routes have been designed to decorate the core pyridine ring with common substituents such as a carbonyl group (*ortho*-aldehyde), an alkoxide (*para*-MeO), and an aromatic ring (*ortho*-pyridine).^12–21^ Yet, the installation of these functionalities *via* C–H activation is not straightforward since it requires several functional group interconversions (Scheme 1B).

**Scheme 1:**
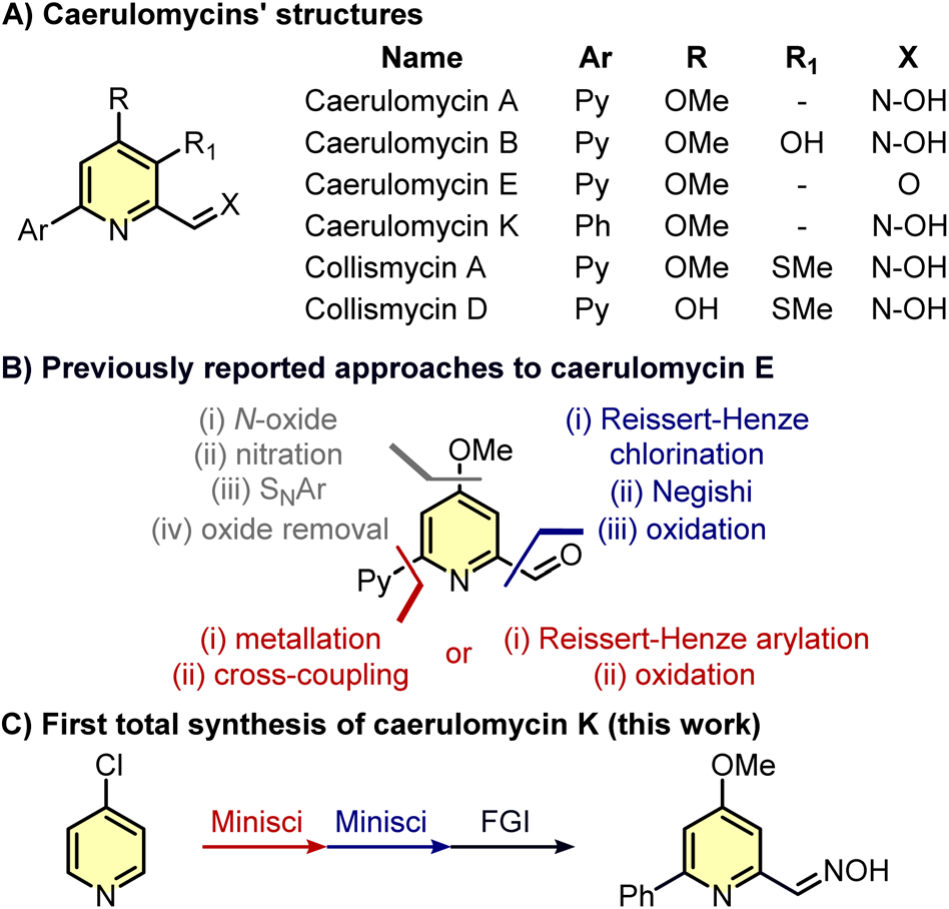
Structures and synthetic routes to caerulomycins.

Specifically, the installation of the methoxy group can require four steps: formation of the N-oxide with an oxidant, nitration with concentrated H_2_SO_4_, nucleophilic aromatic substitution (S_N_Ar) with MeONa, and removal of the oxide with Ac_2_O.^18,19^ The insertion of an *ortho*-pyridine group is usually more rapid but requires the use of Grignard reagents or prefunctionalized 2-bromopyridines.^15,16^ Finally, the insertion of the carbonyl group is achieved by oxidation of a methyl group whose installation has been obtained only with a halogen (Cl or Br) already placed in *ortho*-position.^18,21^ Given the recent advancements in selective C–H functionalizations of pyridines,^4,5^ it would be expected that alternative strategies should now allow a faster synthesis of caerulomycins. Herein, we report our efforts to rapidly convert a cheap monosubstituted pyridine into caerulomycin K, a recently isolated alkaloid whose total synthesis has never been reported before.

Our investigation began with the design of a synthetic route that would furnish caerulomycin K in a few steps using two C–H activations, thus avoiding highly pre-functionalized starting materials. In an initial retrosynthetic approach, we imagined that the aldoxime group could be derived from a methyl group, as reported by Quéguiner and co-workers, thus leading to trifunctionalized pyridine I (Scheme 2A).^18^ At this point, we envisaged that I could be accessed by a selective difunctionalization of pyridyl bis-phosphonium salt III*via* two sequential ligand-coupling (LC) reactions, that is formal S_N_Ar reactions where a phosphonium group is replaced by opportune nucleophiles.^22–24^ Salt III could then be obtained from 2-phenylpyridine 1*via* two consecutive C–P bond formation reactions, in analogy with a rare example of a pyridyl bis-phosphonium salt (Scheme 2B).^25,26^

**Scheme 2:**
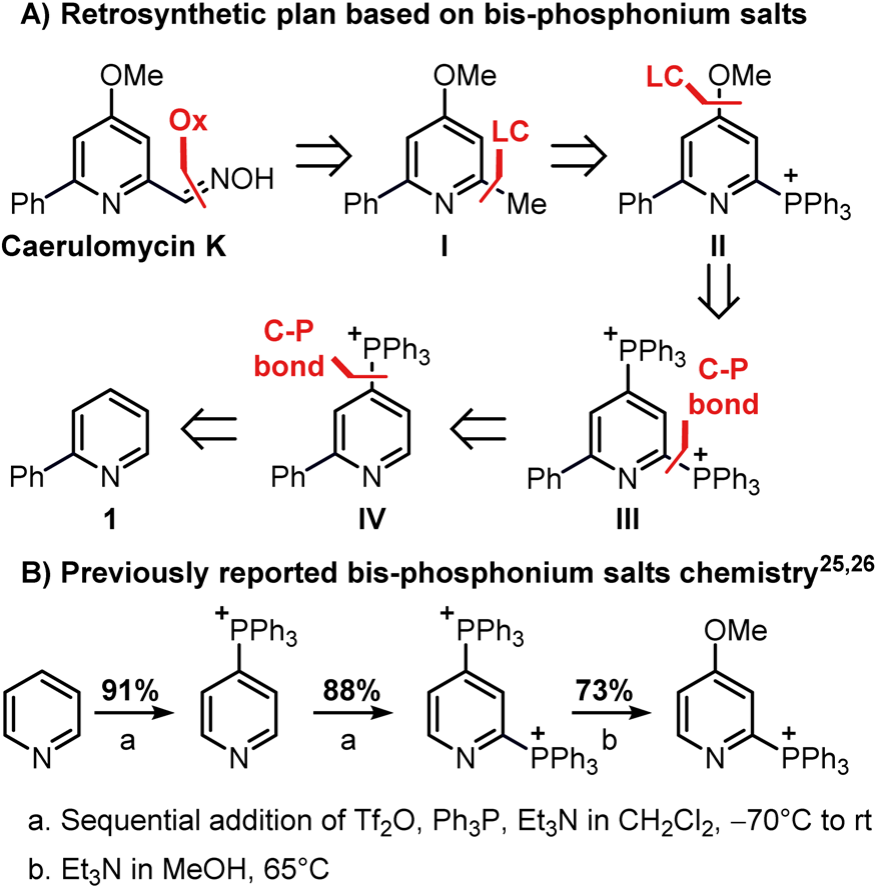
Reactivity of bis-phosphonium salts and application in the planned retrosynthesis of caerulomycin K. The triflate anion is not reported for clarity.

This strategy would provide the desired product in 5 steps, whereas the ionic nature of most intermediates would reduce the need for column chromatography. Moreover, considering the wide versatility of ligand-coupling reactions,^25,26^III would be a strategic intermediate for the synthesis of libraries of trifunctionalized pyridines by simply changing the order and the nature of the added nucleophiles. In the laboratory, 1 was dissolved in dichloromethane and cooled down to −78 °C, before sequentially adding Tf_2_O, Ph_3_P, and DBU (Scheme 3). In agreement with McNally's work,^27^ upon workup, 2 was easily precipitated out as a white powder from cold ether (88% isolated yield). Notably, the Ph_3_P addition occurs almost exclusively at the *para*-position (due to stereoelectronic reasons), thus no regioselectivity problems are encountered during this reaction.^28^ This was also confirmed by ^31^P NMR, with only a sharp singlet observed at 23.01 ppm. Repeating the procedure using now 2 as the starting material, the reaction crude revealed two new signals of similar intensity at 23.55 ppm and 15.37 ppm.

**Scheme 3:**
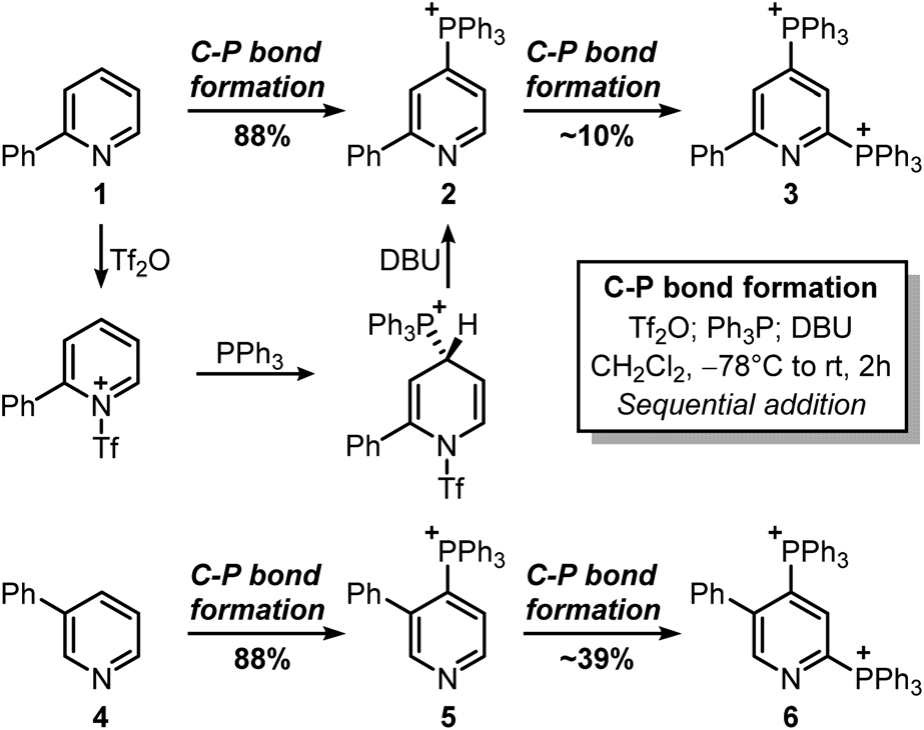
Attempted bis-phosphonium salts synthesis. The triflate anion is not reported for clarity.

These signals were respectively assigned to the *para*- and *ortho*-phosphine of bis-phosphonium bis-triflate 3. However, the conversion was only modest by ^31^P NMR, with significant unreacted 2 and Ph_3_PO observed in the reaction mixture. The failure of the second C–P bond formation was attributed to a problematic N-activation since 2 should be less nucleophilic than 1 due to its cationic nature. This was confirmed using phosphonium 5, obtained in good yield from 3-phenylpyridine 4: moving away the phenyl ring from the *ortho*- to the *meta*-position improved the second C–P bond formation (5 less sterically encumbered than 2), yet not to a significant extent due to electronic reasons. Indeed, bis-phosphonium 6 was found as a minor component in ^31^P NMR spectrum of the reaction crude (signals at 21.93 ppm and 17.08 ppm, with a ^3^*J*_P–P_ = 6.0 Hz). Deuterodephosphination^29^ of this reaction mixture further confirmed the poor conversion, with the isolated pyridine showing almost quantitative *d*-incorporation in *para*-position but a limited deuteration on the C6-site (Scheme 4).

**Scheme 4:**
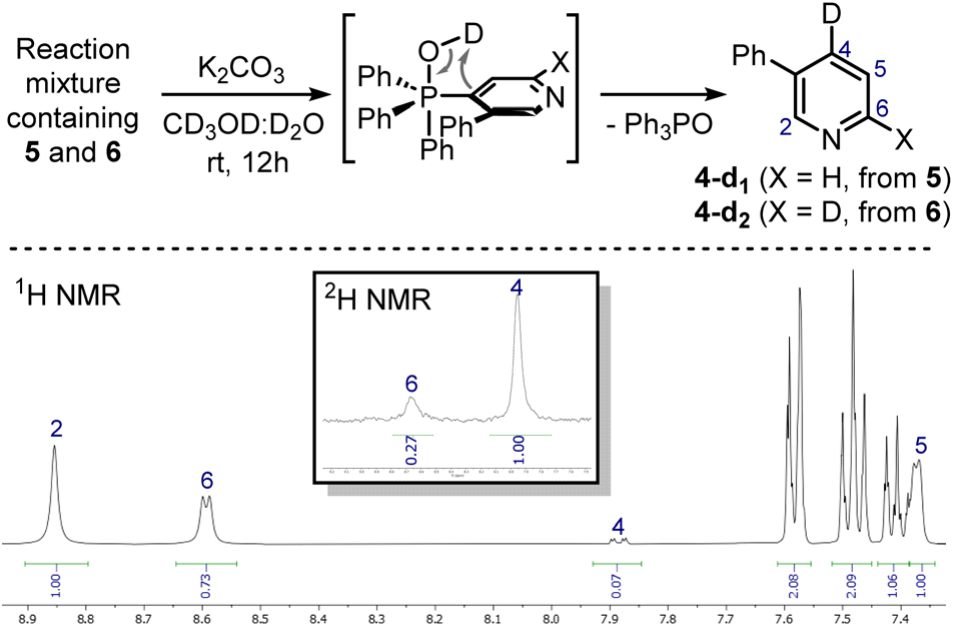
Deuterodephosphination of the 5/6 mixture (top) and ^1^H NMR and ^2^H NMR spectra (CDCl_3_, 400 MHz) of the isolated mixture of 4-d_1_/4-d_2_ (bottom). Deuterium incorporation was determined by the relative integration of the signals of hydrogens on the pyridine ring.

Finally, attempts to use a more nucleophilic phosphine (*i.e.* (4-anisyl)_3_P) did not improve the C–P bond formation, and neither did the use of it as the first installed phosphine (see ESI†). Indeed, the use of (4-anisyl)_3_P mainly resulted in the formation of the corresponding phosphine oxide, as expected for electron-rich phosphines. Given the problematic separation of salts 2 and 3 and the modest conversion observed in the second step, we decided to perform one C–P bond formation at a time. Treatment of 2 with MeONa in dichloromethane gave disubstituted pyridine 7 in 53% ^1^H NMR yield (Scheme 5), although its isolation was complicated by co-eluting Ph_3_PO (the product, together with 1, of competitive protodephosphination). Before performing the second C–P bond formation on 7, the installation of a methyl group using 2 as a model compound was attempted.

**Scheme 5:**
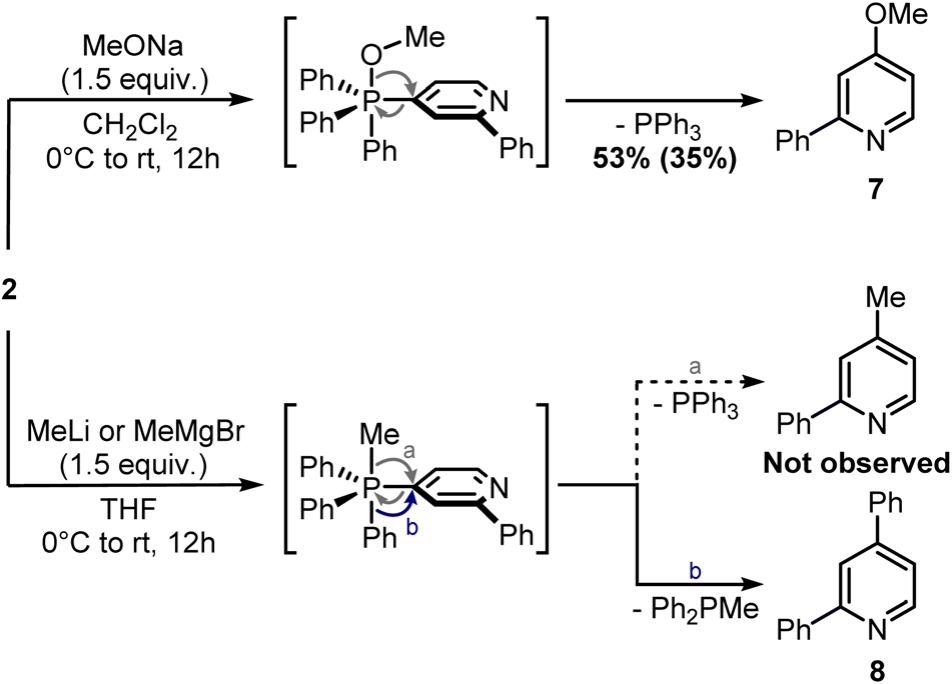
Ligand-coupling reactions with phosphonium salt 2. ^1^H NMR yield determined using CH_2_Br_2_ as internal standard (in brackets, isolated yield).

Indeed, while the replacement of Ph_3_P with chalcogens/pnictogen nucleophiles (–OR, –SR, –NR_2_) is relatively straightforward,^27,30,31^ the installation of alkyl or aryl groups *via* ligand-coupling requires additional manipulations.^32–34^ However, the direct use of organolithium has been shown successful in a couple of cases (*e.g.* ArLi), hence we hoped the use of MeLi or MeMgBr would avoid extra steps.^27^ Unfortunately, treatment of 2 with these organometallics provided equimolar amounts of 2,4-diphenylpyridine 8 and Ph_2_P(O)Me (Scheme 5), the latter observable in the ^1^H NMR spectrum (2.01 ppm, d, ^2^*J*_P–H_ = 13.2 Hz, 3H, Me). This result highlights how, in contrast to alkoxides, the phenyl ring has a higher migration aptitude than a methyl group during the ligand-coupling of the phosphorane intermediate,^35,36^ thus leading to 8 and Ph_2_PMe (then oxidized during the workup). An alternative approach to the use of phosphonium salts would be an *ortho*-halogenation followed by Negishi coupling with MeZnCl, in analogy with the reported synthesis of caerulomycin E.^18^ These halogenations (Reissert–Henze reactions) require the use of N-oxides, easily made upon treatment of pyridines with an oxidant such as H_2_O_2_ or *m*-chloroperbenzoic acid (*m*CPBA).^37^ Initially, using 1-O as a model substrate, activation with Tf_2_O and bromination with tetra-*n*-butylammonium bromide (TBAB) was attempted, in analogy with the *ortho*-bromination of quinolines reported by Baran and co-workers (Scheme 6).^38^ However, no desired product was observed, but only a mixture of brominated pyridines, probably due to some Br_2_ formed from the residual oxidant (*m*CPBA) still present in 1-O. In contrast to pyridines, the successful *ortho*-bromination observed by Baran for quinolines reflects the lower loss in resonance stabilization typical of bicyclic aromatics (*i.e.* naphthalene *vs.* benzene). Chlorination of 1-O with POCl_3_ gave better results (9 isolated in 55% yield), but the need for harsh conditions (neat POCl_3_ refluxing at 106 °C) somehow defeated our original purpose for a short and mild synthesis, thus a completely different strategy was considered.

**Scheme 6:**
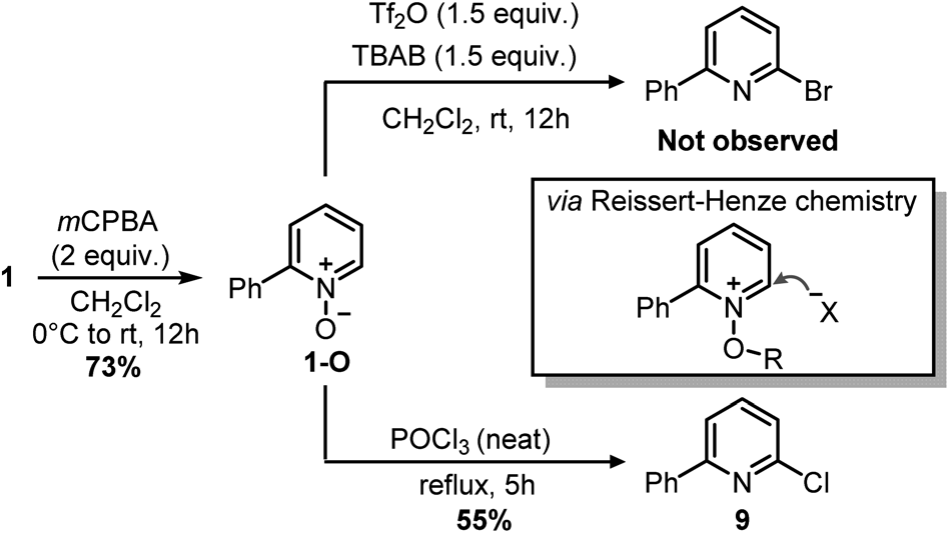
*ortho*-Halogenations with 1-O.

Minisci-type chemistry is an excellent method for *ortho*-functionalizations of pyridines, especially employing nucleophilic carbon-based radicals (ideal for the synthesis of caerulomycin K).^39,40^ Moreover, starting with a 4-substituted pyridine would prevent regioselectivity issues (C2 *vs.* C6) typical of unsymmetrical starting materials. For the *ortho*-arylation of pyridines, Baran and others have shown how aryl boronic esters, in combination with AgNO_3_, Na_2_S_2_O_8_, and TFA, are excellent aryl radical precursors.^41,42^ To install a carbonyl group, Angeles, Yeung, and colleagues have used 1,3,5-trioxanes as an aldehyde equivalent in Minisci-type carbonylation of pyridines.^43^ Based on this precedent, a Minisci arylation of 4-chloropyridine 10 was performed (Scheme 7). In this case, an excess of phenylboronic acid (1.5 equiv.) was needed to compensate for competitive protodeboronation, whereas a higher loading of AgNO_3_ allowed the isolation of product 11 in 56% yield. A second Minisci reaction was then performed on this pyridine using 1,3,5-trioxane in the presence of (*n*Bu_4_N)_2_S_2_O_8_. A successful *ortho*-alkylation gave product 12 in 50% yield (a value in agreement with previous reports),^44–47^ whereas a subsequent nucleophilic aromatic substitution allowed to access trifunctionalized pyridine 13 almost quantitatively. It has to be noted that starting from 4-methoxypyridine 14 would shorten the synthesis, but the electron-donating effect of the methoxy group will negatively affect both steps since Minisci reactions are based on the addition of nucleophilic radicals. The final conversion of 13 into caerulomycin K was achieved in a one-pot procedure by treatment with HCl (to reveal the aldehyde functionality), followed by condensation with NH_2_OH. Importantly, 12 could be directly converted into caerulomycin K without the need for isolation of 13, further simplifying the synthesis (route in green). Therefore, this three-step total synthesis (overall yield of 10%) represents the first synthesis of caerulomycin K as well as a potential alternative to the synthesis of caerulomycins.

**Scheme 7:**
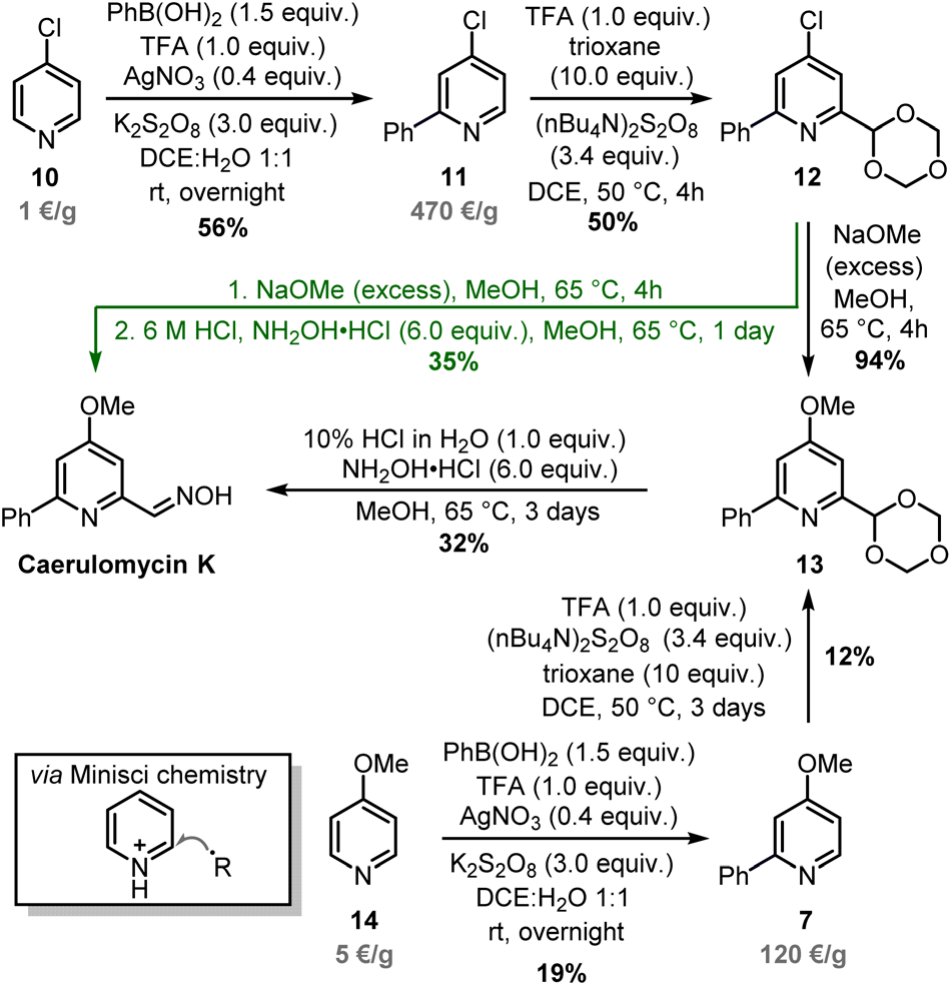
*ortho*-Functionalization of 10 and total synthesis of caerulomycin K.

In conclusion, the first total synthesis of caerulomycin K has been reported. Starting from monofunctionalized pyridines, the first strategy looked at a double C–H activation by means of phosphonium chemistry. However, a poor conversion of the second C–P bond formation and a problematic *ortho*-methylation, including *via* halogenation, prompted the search for a better alternative. This was achieved by sequential Minisci *ortho*-arylation and *ortho*-alkylation, with the latter converted in one pot into the desired oxime. Compared to previously reported caerulomycin syntheses, this novel approach does not require highly pre-functionalized starting materials.

## Conflicts of interest

There are no conflicts to declare.

## Supplementary Material

RA-014-D4RA00589A-s001

RA-014-D4RA00589A-s002
