# Supplementary material for: First total synthesis of caerulomycin K: a case study on selective, multiple C–H functionalizations of pyridines
Source: RSC Adv. 2024 Feb 13;14(8):5542–6. doi: 10.1039/d4ra00589a (PMC10862659; doi:10.1039/d4ra00589a)
Supplement: RA-014-D4RA00589A-s001 [file RA-014-D4RA00589A-s001.pdf]

Electronic Supplementary Material (ESI) for RSC Advances.
This journal is © The Royal Society of Chemistry 2024

Compound Characterization Checklist

- Triphenyl(2-phenylpyridin-4-yl)phosphonium trifluoromethanesulfonate (2)

This known compound was isolated and characterized by ^{1}H NMR and ^{31}P NMR, as reported in the literature.

- Triphenyl(3-phenylpyridin-4-yl)phosphonium trifluoromethanesulfonate (5)

This known compound was isolated and characterized by ^{1}H NMR and ^{31}P NMR, as reported in the literature.

- 4-methoxy-2-phenylpyridine (7)

This known compound was isolated and characterized by ^{1}H NMR, as reported in the literature.

- 2-phenylpyridine 1-oxide (1-O)

This known compound was isolated and characterized by ^{1}H NMR, as reported in the literature.

- 5-bromo-2-phenylpyridine

This known compound was isolated and characterized by ^{1}H NMR, as reported in the literature.

- 4-bromo-2-phenylpyridine

This known compound was isolated and characterized by ^{1}H NMR, as reported in the literature.

- 3-bromo-2-phenylpyridine

This known compound was isolated and characterized by ^{1}H NMR, as reported in the literature.

- 2-chloro-6-phenylpyridine (9)

This known compound was isolated and characterized by ^{1}H NMR, as reported in the literature.

- 4-chloro-2-phenylpyridine (11)

This known compound was isolated and characterized by ^{1}H NMR, as reported in the literature.

- 4-chloro-2-phenyl-6-(1,3,5-trioxan-2-yl)pyridine (12)

This novel compound was isolated and characterized by ^{1}H NMR and ^{13}C NMR, as well as HRMS.

- 4-methoxy-2-phenyl-6-(1,3,5-trioxan-2-yl)pyridine (13)

This novel compound was isolated and characterized by ^{1}H NMR and ^{13}C NMR, as well as HRMS.

- Caerulomycin K

This known compound was isolated and characterized by ^{1}H NMR and ^{13}C NMR, as reported in the literature.
