# Supplementary material for: First total synthesis of caerulomycin K: a case study on selective, multiple C–H functionalizations of pyridines
Source: RSC Adv. 2024 Feb 13;14(8):5542–6. doi: 10.1039/d4ra00589a (PMC10862659; doi:10.1039/d4ra00589a)
Supplement: RA-014-D4RA00589A-s002 [file RA-014-D4RA00589A-s002.pdf]

Electronic Supplementary Material (ESI) for RSC Advances.
This journal is © The Royal Society of Chemistry 2024

Supporting Information for

First Total Synthesis of Caerulomycin K: A Case Study on Selective, Multiple C-H Functionalizations of Pyridines

Alessandro Dimasi+,[a] Mattia Failla+,[a] Arianna Montoli,[a] Andrea Citarella,[a] Paolo Ronchi,[b] Daniele Passarella[a] and Valerio Fasano*[a]

[a] A. Dimasi,+ M. Failla,+ A. Montoli, Dr. A. Citarella, Prof. D. Passarella, Dr. V. Fasano
Department of Chemistry
Università degli Studi di Milano
Via Camillo Golgi, 19, 20133 Milano (Italy)
E-mail: valerio.fasano@unimi.it
www.fasanolab.com

[b] Dr. P. Ronchi
Chemistry Research and Drug Design
Chiesi Farmaceutici S.p.A
Largo Belloli 11/a, 43126 Parma (Italy)

TABLE OF CONTENTS

1. MATERIALS AND GENERAL METHODS 2
1.1. General considerations 2
1.2. Naming of Compounds 2
2. EXPERIMENTAL DATA 3
2.1. Synthesis of phosphonium salts 3
General Procedure (GP-A) for the synthesis of phosphonium salts 3
Synthesis of triphenyl(2-phenylpyridin-4-yl)phosphonium trifluoromethanesulfonate (2) 3
Synthesis of (6-phenylpyridine-2,4-diyl)bis(triphenylphosphonium) ditrifluoromethanesulfonate (3) 4
Synthesis of triphenyl(3-phenylpyridin-4-yl)phosphonium trifluoromethanesulfonate (5) 5
Synthesis of (5-phenylpyridine-2,4-diyl)bis(triphenylphosphonium) ditrifluoromethanesulfonate (6) 5
Attempted formation of tris(4-methoxyphenyl)(2-phenylpyridin-4-yl)phosphonium trifluoromethanesulfonate 6
2.2. Deuterodephosphination experiment 7
2.3. Ligand-Coupling reactions 10
General Procedure (GP-B) for Ligand-Coupling reactions 10
Synthesis of 4-methoxy-2-phenylpyridine (7) from phosphonium salt 2 10
Attempted methylation 11
2.4. *Ortho*-halogenation experiments *via* Reissert-Henze chemistry 12
Synthesis of 2-phenylpyridine 1-oxide (1-O) 12
Attempted bromination of 1-O 13
Synthesis of 2-chloro-6-phenylpyridine (9) *via* chlorination of 1-O 14
2.5. *Ortho*-alkylations *via* Minisci chemistry 14
Synthesis of 4-chloro-2-phenylpyridine (11) from 4-chloropyridine (10) 14
Synthesis of 4-chloro-2-phenyl-6-(1,3,5-trioxan-2-yl)pyridine (12) from 11 15
Synthesis of 4-methoxy-2-phenyl-6-(1,3,5-trioxan-2-yl)pyridine (13) from 12 15
Synthesis of 4-methoxy-2-phenylpyridine (7) from 4-methoxypyridine (14) 16
Synthesis of 4-methoxy-2-phenyl-6-(1,3,5-trioxan-2-yl)pyridine (13) from 7 16
Synthesis of caerulomycin K from 13 17
Synthesis of caerulomycin K from 12 17
3. SPECTROSCOPIC DATA 18
4. REFERENCES 28

1. MATERIALS AND GENERAL METHODS

1.1. General considerations

Unless stated, all starting materials and anhydrous solvents were obtained from commercial sources and used without purification. Reactions were carried out under an inert atmosphere of nitrogen unless stated. Reaction progress was monitored by TLC, with ^{1}H NMR or LC-MS analyses taken from reaction samples. Column chromatography was performed on silica gel (230-400 mesh) or automated Isolera One Flash Chromatography (Biotage). NMR spectra were recorded with a Bruker AV-400 spectrometer (400 MHz ^{1}H; 101 MHz ^{13}C; 162 MHz ^{31}P). ^{1}H NMR chemical shifts are reported in ppm relative to protio impurities in the deuterated solvents and reported as follow: chemical shift (multiplicity, coupling constants, number of protons). ^{13}C NMR chemical shifts are reported in ppm using the solvent resonance. ^{31}P NMR spectra were recorded using H_3PO_4 (85%) as an external reference. Coupling constants J are given in Hertz (Hz), while the multiplicity of the signals are indicated as “s”, “d”, “t”, “q”, “pent”, “sept” or “m” for singlet, doublet, triplet, quartet, pentet, septet or multiplet, respectively. Mass spectra were recorded on a Waters QTOF mass spectrometer.

1.2. Naming of Compounds

Compound names are those generated by ChemDraw Professional 20.0 software (PerkinElmer), following the IUPAC nomenclature.

2. EXPERIMENTAL DATA

2.1. Synthesis of phosphonium salts

General Procedure (GP-A) for the synthesis of phosphonium salts

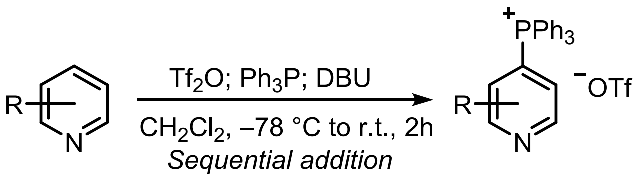

The procedure has been adapted from the literature.[1] A round bottom flask equipped with a stir bar was charged with the heterocycle (1.0 equiv.) and placed under a nitrogen atmosphere. Then, CH_2Cl_2 (0.2 M) was added, and the reaction vessel cooled to –78 °C, followed by the dropwise addition of Tf_2O (1.1 equiv.). The reaction mixture was stirred at –78 °C for 30 minutes, followed by the addition of PPh_3 (1.1 equiv.), and, after 30 minutes, of DBU (1.0 equiv.). After the last addition, the cooling bath was removed, and the reaction was allowed to warm to room temperature while stirring (approximately 15-30 minutes). The reaction mixture was thus quenched with H_2O (approximately the same volume as CH_2Cl_2), the layers separated, and the aqueous phase was extracted 3 times with CH_2Cl_2. The combined organic layers were dried over anhydrous Na_2SO_4, filtered and concentrated under reduced pressure to approximately 2-10 mL. An excess of chilled Et_2O (0 °C) was added to the concentrated solution as the solution started to solidify. The resulting suspension was filtered, and the solid was washed with chilled Et_2O (0 °C) and dried *in vacuo* to provide the pure phosphonium salt.

Synthesis of triphenyl(2-phenylpyridin-4-yl)phosphonium trifluoromethanesulfonate (2)

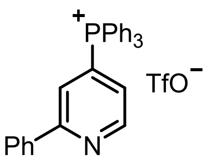

2

Prepared according to the GP-A using 2-phenylpyridine **1** (429 µL, 3.0 mmol, 1.0 equiv.), Tf_2O (555 µL, 3.3 mmol, 1.1 equiv), PPh_3 (866 mg, 3.3 mmol, 1.1 equiv), DBU (448 µL, 3.0 mmol, 1.0 equiv.) and CH_2Cl_2 (15.0 mL). After purification, compound **2** was isolated as a white solid (1.495 g, 2.6 mmol, 88% yield). ^1H NMR (400 MHz, $CDCl_3$) δ: 9.09 (app t, J = 5.0 Hz, 1H), 7.98 – 7.88 (m, 5H), 7.86 – 7.79 (m, 7H), 7.77 – 7.68 (m, 6H), 7.56 (ddd, J = 12.8, 5.1, 1.6 Hz, 1H), 7.52 – 7.46 (m, 3H) ppm. ^{31}P NMR (162 MHz, $CDCl_3$) δ 23.01 ppm. The data are in agreement with those reported in the literature.[1]

See spectrum

Synthesis of (6-phenylpyridine-2,4-diyl)bis(triphenylphosphonium) ditrifluoromethanesulfonate (3)

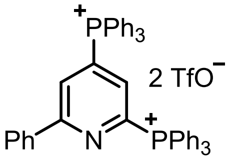

3

Prepared according to GP-A using phosphonium **2** (85 mg, 0.15 mmol, 1.0 equiv.), Tf_2O (28 µL, 0.17 mmol, 1.1 equiv.), PPh_3 (43 mg, 0.17 mmol, 1.1 equiv.), DBU (22 µL, 0.15 mmol, 1.0 equiv.) and CH_2Cl_2 (0.75 mL). After work up, the reaction crude revealed two new signals of similar intensity at 23.55 ppm and 15.37 ppm (^{31}P NMR). These signals were respectively assigned to the *para*- and *ortho*-phosphine of bis-phosphonium bis-triflate **3**. However, isolation of **3** was complicated by the significant amount of unreacted **2** and Ph_3PO (29.56 ppm) observed in the reaction mixture.[2]

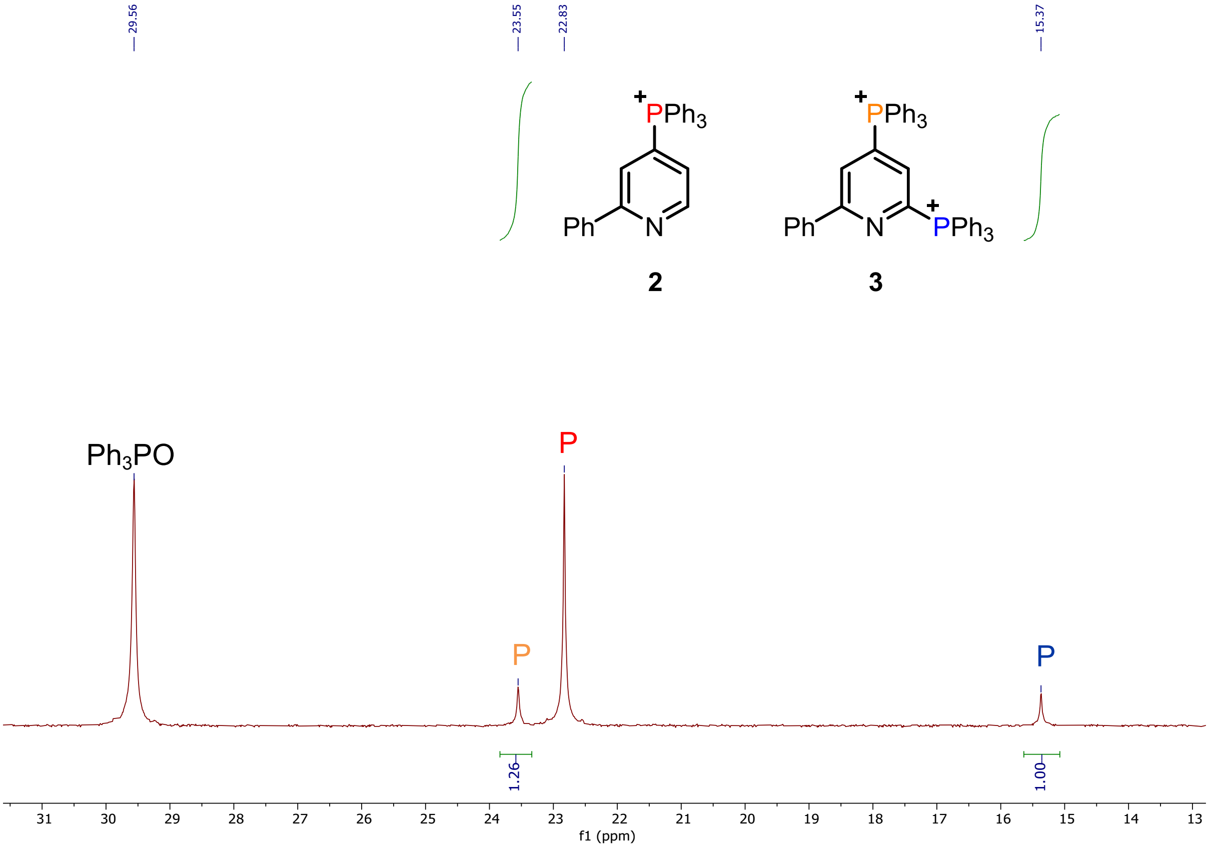

Figure S1. ^{31}P NMR spectrum of the reaction crude containing **3**.

Synthesis of triphenyl(3-phenylpyridin-4-yl)phosphonium trifluoromethanesulfonate (5)

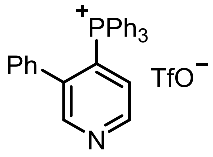

5

Prepared according to GP-A using 3-phenylpyridine **4** (143 µL, 1.0 mmol, 1.0 equiv.), Tf_2O (185 µL, 1.1 mmol, 1.1 equiv.), PPh_3 (288 mg, 1.1 mmol, 1.1 equiv.), DBU (149 µL, 1.0 mmol, 1.0 equiv.) and CH_2Cl_2 (5.0 mL). After purification, compound **5** was isolated as a white solid (498 mg, 0.88 mmol, 88% yield). ^{1}H NMR (400 MHz, $CDCl_3$) δ: 8.97 (d, J = 5.2 Hz, 1H), 8.76 (d, J = 6.8 Hz, 1H), 7.84 – 7.76 (m, 3H), 7.72 – 7.54 (m, 12H), 7.51 (dd, J = 14.9, 5.2 Hz, 1H), 7.12 (t, J = 7.6 Hz, 1H), 6.92 (t, J = 7.6 Hz, 2H), 6.73 (d, J = 7.6 Hz, 2H) ppm. ^{31}P NMR (162 MHz, $CDCl_3$) δ 21.60 ppm. The data are in agreement with those reported in the literature.[1]

See spectrum

Synthesis of (5-phenylpyridine-2,4-diyl)bis(triphenylphosphonium) ditrifluoromethanesulfonate (6)

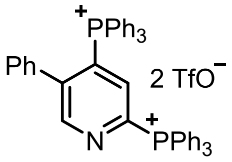

6

Prepared according to GP-A using phosphonium **5** (85 mg, 0.15 mmol, 1.0 equiv.), Tf_2O (28 µL, 0.17 mmol, 1.1 equiv.), PPh_3 (43 mg, 0.17 mmol, 1.1 equiv.), DBU (22 µL, 0.15 mmol, 1.0 equiv.) and CH_2Cl_2 (0.75 mL). After work up, the reaction crude revealed two new signals of similar intensity at 21.93 ppm and 17.08 ppm, with a $^3J_{P\text{-}P}$ = 6.0 Hz (^{31}P NMR). These signals were respectively assigned to the *para*- and *ortho*-phosphine of bis-phosphonium bis-triflate **6**. However, isolation of **6** was complicated by the significant amount of unreacted **5** and Ph_3PO (29.67 ppm) observed in the reaction mixture.[2]

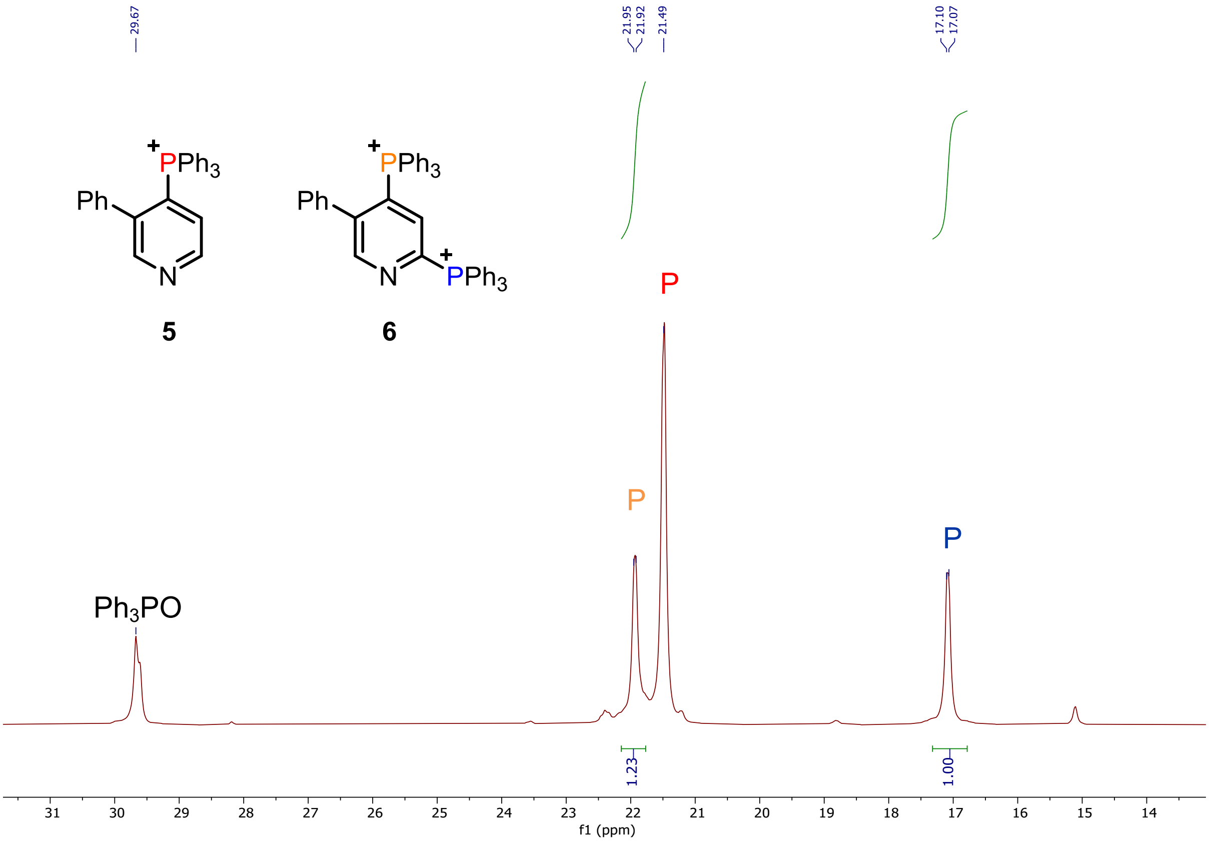

Figure S2. ^{31}P NMR spectrum of the reaction crude containing **5**.

Attempted formation of tris(4-methoxyphenyl)(2-phenylpyridin-4-yl)phosphonium trifluoromethanesulfonate

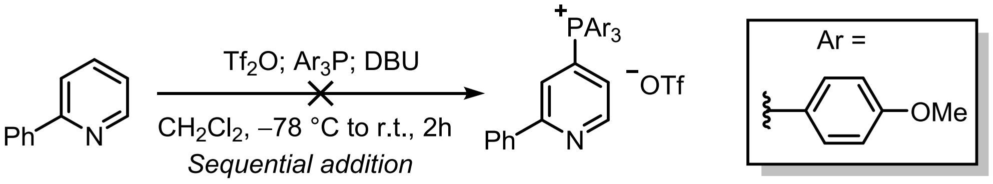

Attempted preparation according to GP-A using 2-phenylpyridine **1** (143 μL, 1.0 mmol, 1.0 equiv.), Tf_2O (185 μL, 1.1 mmol, 1.1 equiv.), (4-anisyl)$_3$P (388 mg, 1.7 mmol, 1.1 equiv.), DBU (149 μL, 1.0 mmol, 1.0 equiv.) and CH_2Cl_2 (5.0 mL). After purification, ^{31}P NMR analysis of the reaction crude revealed two new peaks at 20.80 ppm and 13.97 ppm which we tentatively assign to the addition of Ar_3P to *para*- and *ortho*-position, respectively. However, significant oxidation of (4-anisyl)$_3$P to the corresponding oxide was observed, as expected for this electron-rich phosphine (signal at 28.87 ppm).[3] Similar results were obtained repeating the procedure using **2** as substrate.

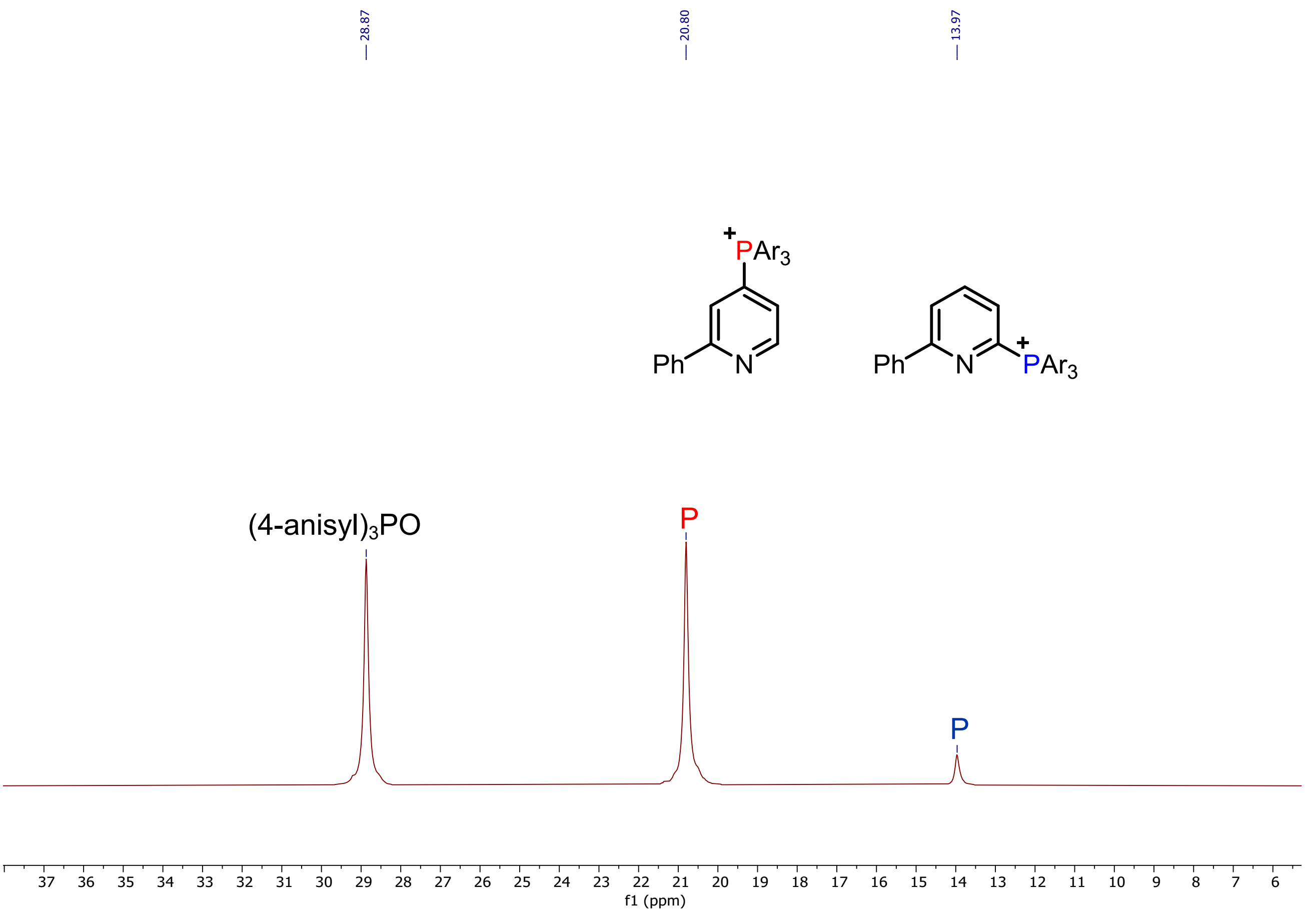

Figure S3. ^{31}P NMR spectrum of the attempted reaction using $(4\text{-anisyl})_3P$.

2.2. Deuterodephosphination experiment

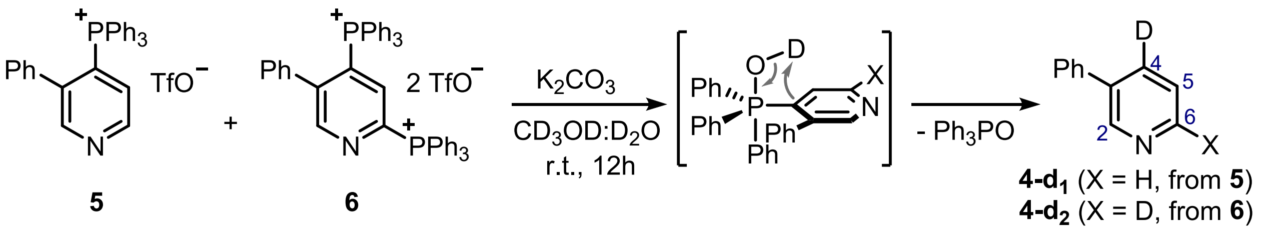

An oven dried round bottom flask equipped with a stir bar was charged with the reaction crude containing **5** and **6** (85 mg in total), K_2CO_3 (31 mg, 0.20 mmol, excess), and placed under a nitrogen atmosphere. CD_3OD:D_2O 9:1 (0.50 mL, 0.3 M) was added at room temperature and the reaction was stirred for 2 hours. The reaction mixture was diluted with CH_2Cl_2 (1 mL) and the organic layer separated, dried over Na_2SO_4, filtered, and concentrated *in vacuo*. The residue was purified by automated column chromatography (hex:EtOAc 90:10 to 0:100), providing deuterated pyridines **4-d$_1$** and **4-d$_2$** as an inseparable mixture.

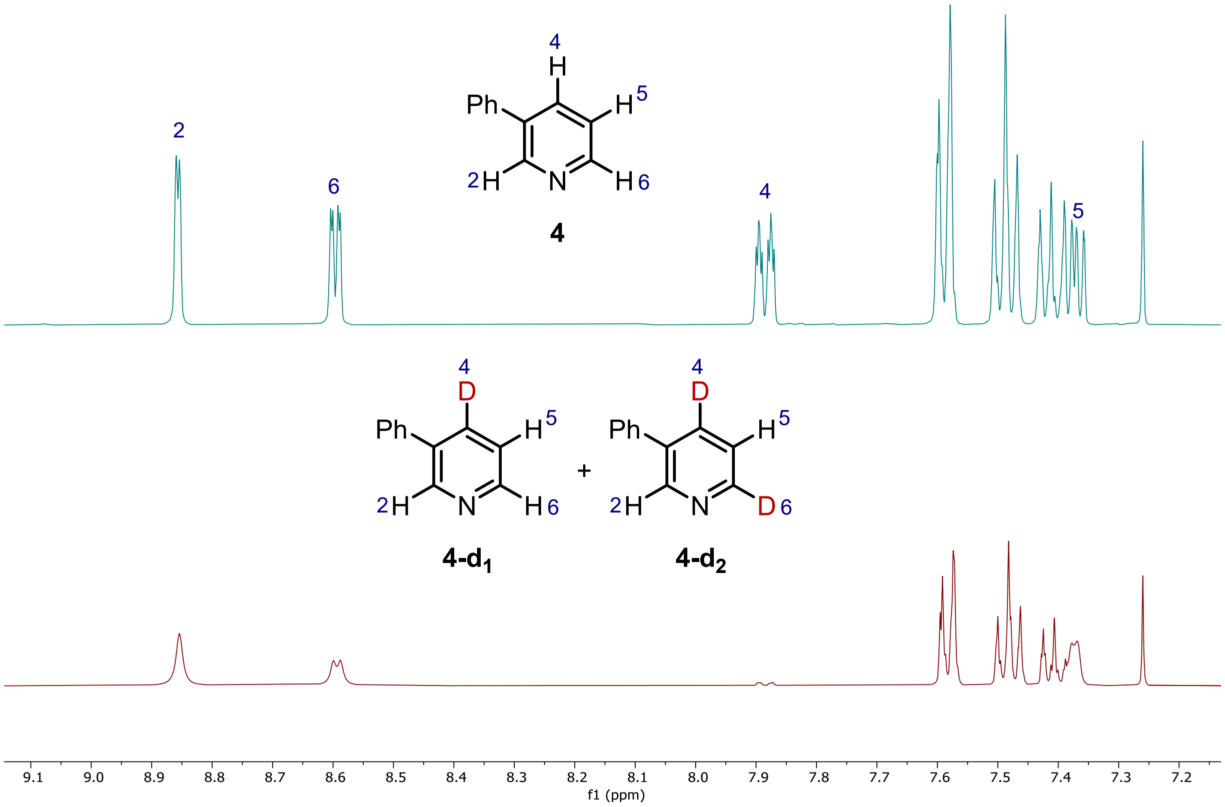

Figure S4. Stacked ^{1}H-NMR spectra of **4-d$_1$**:**4-d$_2$** mixture and **4**, showing how deuterium incorporation in *ortho*- and *para*-position causes less intense signals in the respective positions as well as loss of coupling on the remaining protons.

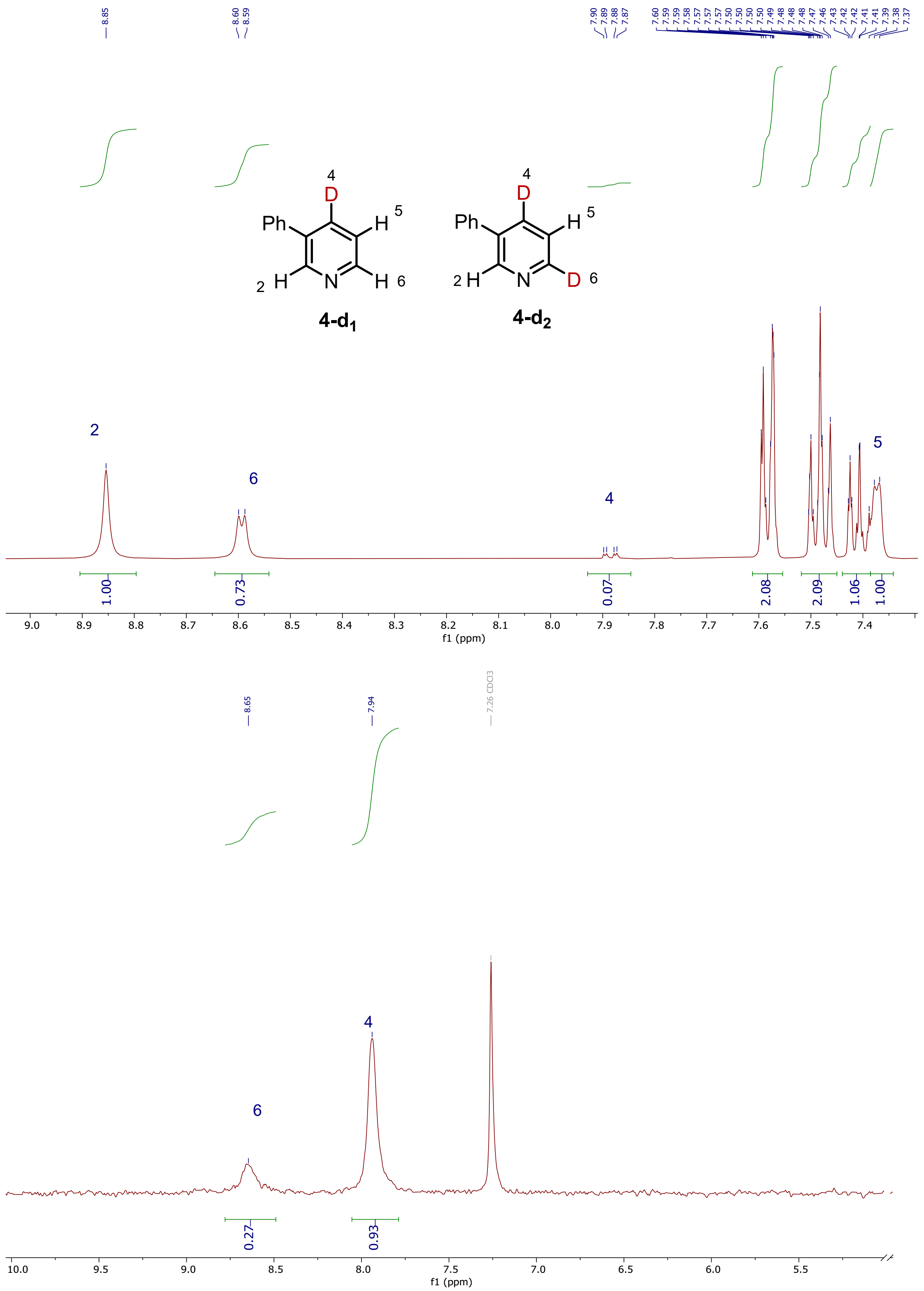

Figure S5. ^{1}H NMR (top) and ^{2}H NMR (bottom) and of the inseparable mixture of **4-d$_1$**/**4-d$_2$** showing 27% deuterium incorporation in *ortho*-position and 93% in *para*-position.

2.3. Ligand-Coupling reactions

General Procedure (GP-B) for Ligand-Coupling reactions

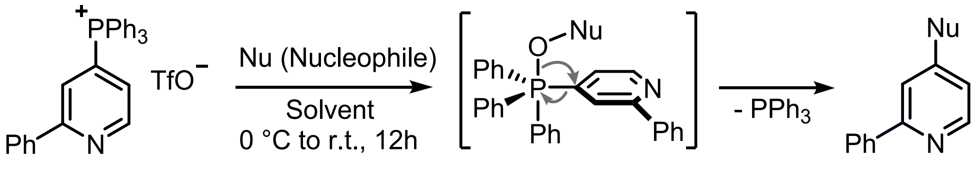

The procedure has been adapted from the literature.[4] An oven dried 10 mL round bottom flask was charged with the phosphonium salt **2** (1.0 equiv.) and subjected to three rapid cycles of vacuum/nitrogen backfill. The solvent (0.2 or 0.5 M) was added, and the solution was stirred at 0 °C. The opportune nucleophile (1.5 equiv.) was then added, leaving the reaction stirring overnight at room temperature. The reaction mixture was quenched with H_2O (approximately the same volume as the solvent), the layers separated, and the aqueous phase was extracted 3 times with CH_2Cl_2. The combined organic layers were dried over anhydrous Na_2SO_4, filtered and concentrated under reduced pressure. The residue was then purified by column chromatography.

Synthesis of 4-methoxy-2-phenylpyridine (7) from phosphonium salt 2

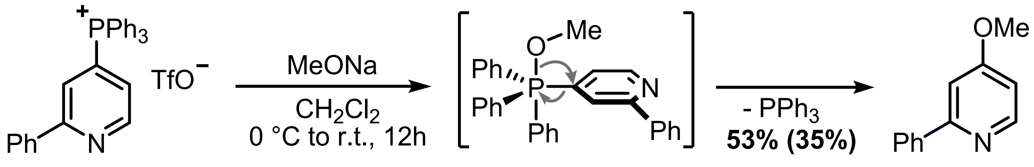

Prepared according to GP-B using phosphonium salt **2** (85 mg, 0.15 mmol, 1.0 equiv.) in CH_2Cl_2 (0.3 mL, 0.2 M) and MeONa (12 mg, 0.23 mmol, 1.5 equiv.). ^{1}H NMR yield using CH_2Br_2 as the internal standard revealed 53% product formation. The residue was purified by automated column chromatography (hex:EtOAc 90:10 to 30:70) to provide product **7** (10 mg, 0.052 mmol) as clear oil in 35% isolated yield (isolation complicated by co-eluting Ph_3PO). ^{1}H NMR (400 MHz, $CDCl_3$) δ: 8.53 (d, *J* = 5.7 Hz, 1H), 7.99 –7.93 (m, 2H), 7.54 –7.39 (m, 3H), 7.24 (d, *J* = 2.4 Hz, 1H), 6.80 (dd, *J* = 5.7, 2.4 Hz, 1H), 3.92 (s, 3H). The data are in agreement with those reported in the literature.[4]

See spectrum

Attempted methylation

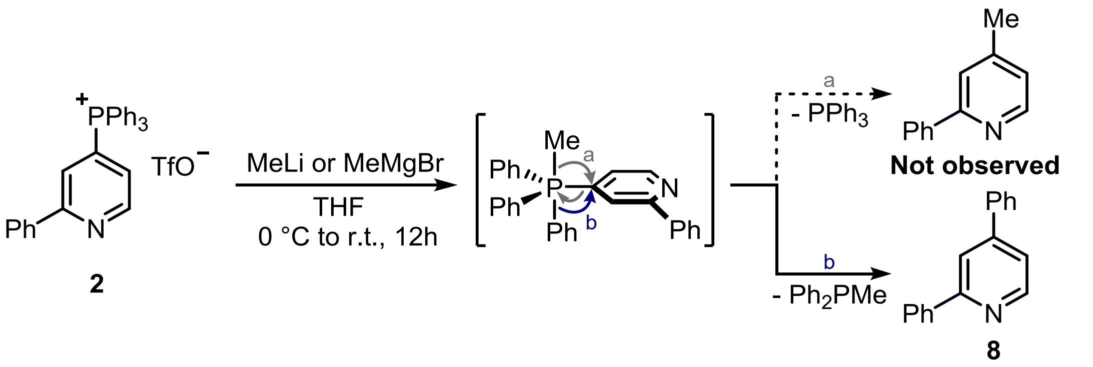

Attempted preparation according to GP-B using phosphonium salt **2** (113 mg, 0.20 mmol, 1.0 equiv.) in THF (1.0 mL, 0.2 M) and MeLi in THF (188 µL, 0.30 mmol, 1.5 equiv.). Performing the addition of this nucleophile at –78 °C did not alter the outcome of the reaction, neither did the use of MeLi·LiBr or MeMgBr. Upon workup, 2,4-diphenylpyridine and Ph_2(Me)PO were observed as major products, the latter clearly visible in the ^{1}H NMR spectrum as a doublet at 2.01 ppm with a *J* = 13.2 Hz and in the ^{31}P NMR spectrum at 29.19 ppm.[5]

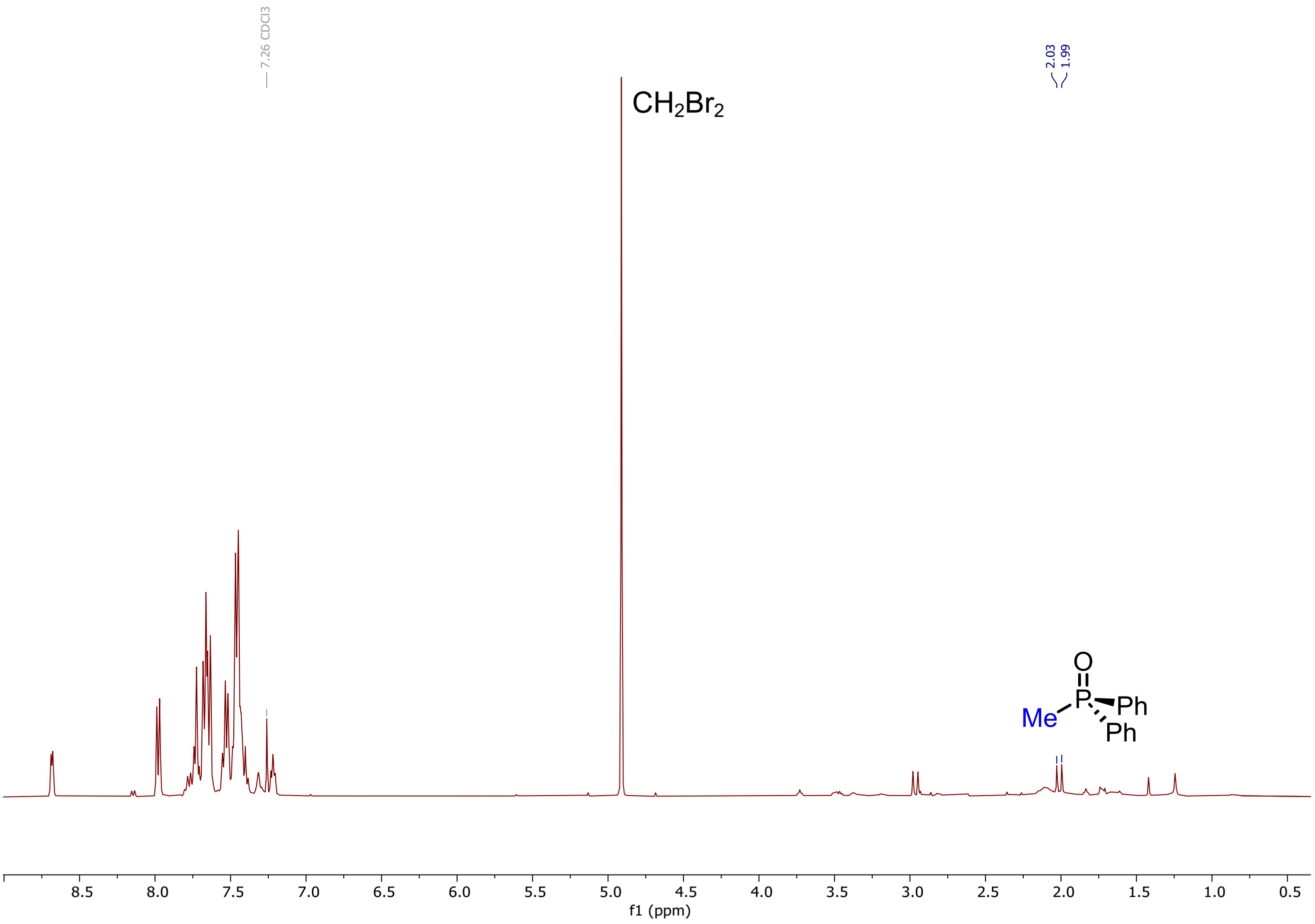

Figure S6. ^{1}H NMR spectrum of the attempted methylation (CH_2Br_2 is the internal standard).

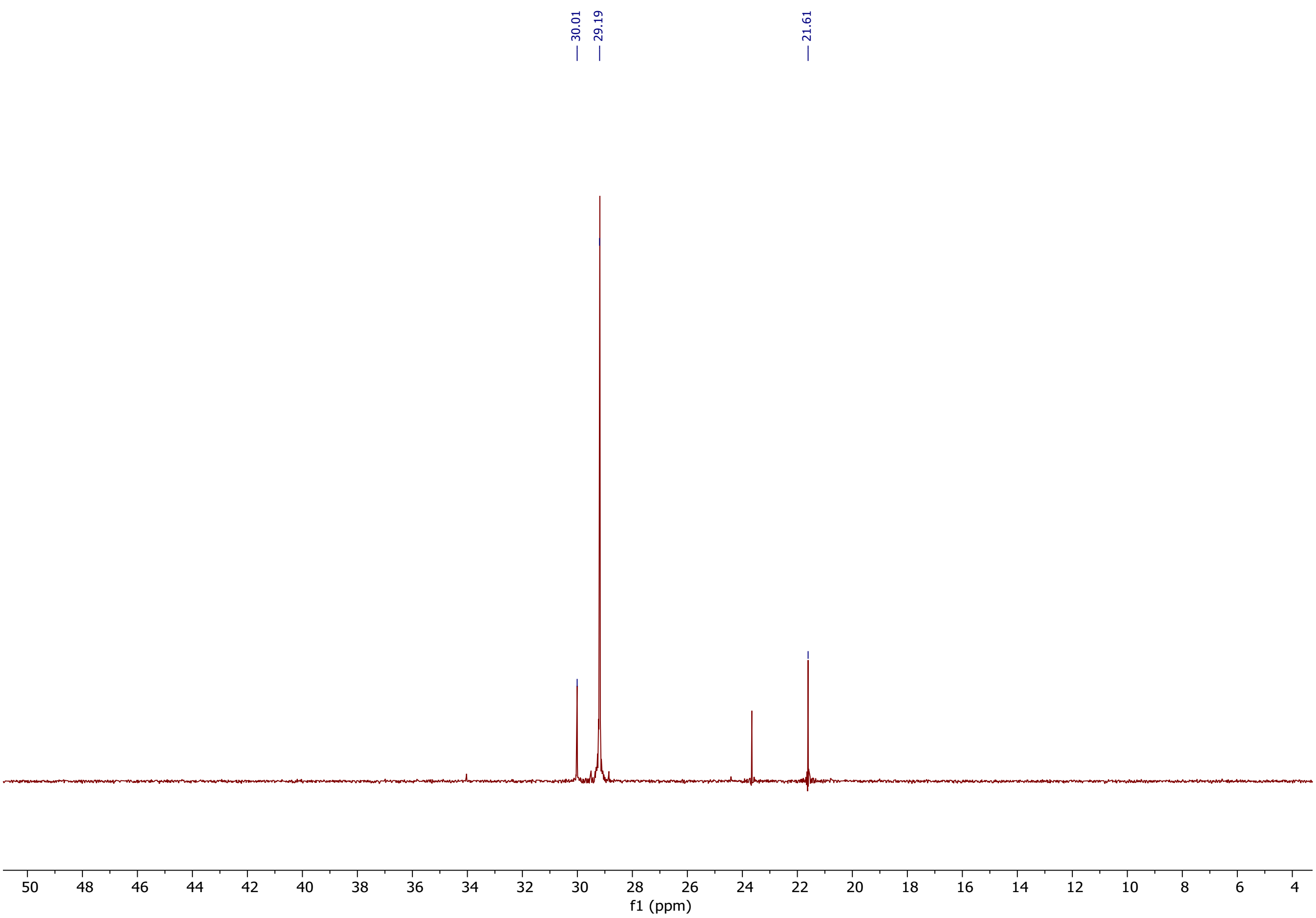

Figure S7. ^{31}P NMR spectrum of the attempted methylation

2.4. *Ortho*-halogenation experiments *via* Reissert-Henze chemistry

Synthesis of 2-phenylpyridine 1-oxide (1-O)

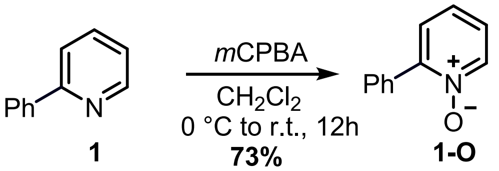

A solution of **1** (143 µL, 1.0 mmol, 1.0 equiv.) in CH_2Cl_2 (2.0 mL, 0.5 M) was cooled down to 0 °C, followed by the addition of *m*CPBA (246 µL, 2.0 mmol, 2.0 equiv.). After leaving the reaction mixture stirring at room temperature overnight, it was diluted with CH_2Cl_2 (6 mL) and washed with KOH 6 N (3x 8 mL). The organic layer was then dried over Na_2SO_4, filtered, and concentrated under reduced pressure, yielding **1-O** as a white solid, used later without any further purification (125.0 mg, 0.730 mmol, 73% yield). ^1H NMR (400 MHz, $CDCl_3$) δ: 8.56 (d, *J* = 6.2 Hz, 1H), 7.85 – 7.78 (m, 2H), 7.55 – 7.44 (m, 5H), 7.38 – 7.35 (m, 1H). The data are in agreement with those reported in the literature.[6]

See spectrum

Attempted bromination of 1-O

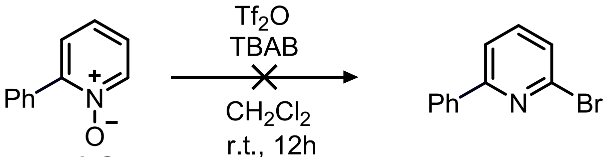

1-O

Tetrabutylammonium bromide [TBAB] (242 mg, 0.75 mmol, 1.5 equiv.) and **1-O** (86 mg, 0.50 mmol, 1.0 equiv.) were dissolved in CH_2Cl_2 (50.0 mL, 0.01 M) for 10 minutes. Then, Tf_2O (126 µL, 0.75 mmol, 1.5 equiv.) was added, leaving the reaction mixture stirring at room temperature overnight. The reaction mixture was then filtrated and concentrated under reduced pressure. The crude product was purified by automated column chromatography (hex:EtOAc 100:0 to 80:20) but no desired product was isolated. Instead, **5-bromo-2-phenylpyridine** (colorless oil, 19 mg, 0.081 mmol, 16% yield), **4-bromo-2-phenylpyridine** (colorless oil, 10 mg, 0.040 mmol, 8% yield), and **3-bromo-2-phenylpyridine** (colorless oil, 5 mg, 0.020 mmol, 4% yield) were isolated, probably due to some Br_2 formed from the residual oxidant (*m*CPBA) still present in **1-O**.

5-bromo-2-phenylpyridine

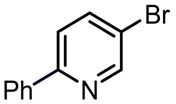

^{1}H NMR (400 MHz, $CDCl_3$) δ: 8.74 (d, J = 2.4 Hz, 1H), 7.96 (dd, J = 8.2, 1.6 Hz, 2H), 7.87 (dd, J = 8.5, 2.4 Hz, 1H), 7.63 (d, J = 8.5, 1H), 7.52 – 7.41 (m, 3H). The data are in agreement with those reported in the literature.[7]

See spectrum

4-bromo-2-phenylpyridine

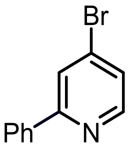

^{1}H NMR (400 MHz, $CDCl_3$) δ: 8.51 (d, J = 5.2 Hz, 1H), 7.97 (dd, J = 8.2, 1.5 Hz, 2H), 7.91 (d, J = 1.8 Hz, 1H), 7.55 – 7.43 (m, 3H), 7.41 (dd, J = 5.2, 1.8 Hz, 1H). The data are in agreement with those reported in the literature.[7]

See spectrum

3-bromo-2-phenylpyridine

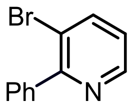

^{1}H NMR (400 MHz, $CDCl_3$) δ: 8.63 (dd, *J* = 4.7, 1.5 Hz, 1H), 8.00 (dd, *J* = 8.1, 1.5 Hz, 1H), 7.68 (dd, *J* = 7.9, 1.7 Hz, 2H), 7.55 – 7.40 (m, 3H), 7.15 (dd, *J* = 8.1, 4.7 Hz, 1H).The data are in agreement with those reported in the literature.[7]

See spectrum

Synthesis of 2-chloro-6-phenylpyridine (9) *via* chlorination of 1-O

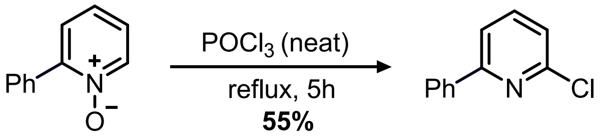

1-O **9**

1-O (34 mg, 0.20 mmol, 1.0 equiv.) was dissolved in $POCl_3$ (3.0 mL, excess) and heated under reflux (106 °C) for 5 hours. The reaction mixture was then concentrated under reduced pressure, followed by the addition of water (5 mL), neutralization with an aqueous solution of K_2CO_3 (5 mL), and extraction with CH_2Cl_2 (3x 10 mL). The reaction crude was then purified by column chromatography (hex:EtOAc 80:20), to afford compound **9** as a yellowish oil (21 mg, 0.11 mol, 55% yield). ^{1}H NMR (400 MHz, $CDCl_3$) δ: 8.00 (dd, *J* = 8.2, 1.5 Hz, 2H), 7.71 (t, *J* = 7.7 Hz, 1H), 7.65 (dd, *J* = 7.7, 1.0 Hz, 1H), 7.52 – 7.41 (m, 3H), 7.26 (dd, *J* = 7.7, 1.0 Hz, 1H) ppm. The data are in agreement with those reported in the literature.[8]

See spectrum

2.5. *Ortho*-alkylations *via* Minisci chemistry

Synthesis of 4-chloro-2-phenylpyridine (11) from 4-chloropyridine (10)

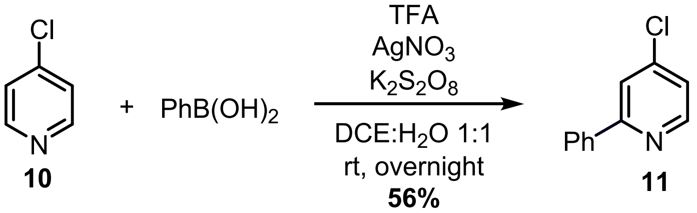

10 **11**

4-chloropyridine hydrochloride (600 mg, 4.0 mmol, 1.0 equiv.) was dissolved in CH_2Cl_2 and washed with saturated $NaHCO_3$. The aqueous phase was extracted with CH_2Cl_2 (3x 15 mL) and the collected organic phases dried over Na_2SO_4, filtered and concentrated under reduced pressure. The resulting 4-chloropyridine **10** was dissolved in 1,2-dichloroethane [DCE] (15.0 mL, 0.27 M), followed by the

addition of trifluoroacetic acid [TFA] (310 µL, 4.00 mmol, 1.0 equiv.) and phenylboronic acid (732 mg, 6.0 mmol, 1.5 equiv.). H_2O (15.0 mL, 0.27 M) was then added, followed by $AgNO_3$ (272 mg, 1.6 mmol, 0.40 equiv.) and $K_2S_2O_8$ (3240 mg, 12.0 mmol, 3.0 equiv.). The reaction was kept at room temperature overnight until completion. Then, the phases were separated and the aqueous phase was extracted with CH_2Cl_2 (2x 15 mL). The collected organic phases were dried over Na_2SO_4, filtered and concentrated *in vacuo*. The crude was purified by column chromatography (CH_2Cl_2:MeOH 95:5) to afford compound **11** as a white solid (425 mg, 2.2 mmol, 56% yield). ^{1}H NMR (400 MHz, $CDCl_3$) δ: 8.59 (d, J = 5.3 Hz, 1H), 7.98 (dd, J = 8.2, 1.6 Hz, 2H), 7.74 (d, J = 1.8 Hz, 1H), 7.52 – 7.40 (m, 3H), 7.25 (dd, J = 5.3, 1.8 Hz, 1H). The data are in agreement with those reported in the literature.[4]

See spectrum

Synthesis of 4-chloro-2-phenyl-6-(1,3,5-trioxan-2-yl)pyridine (12) from 11

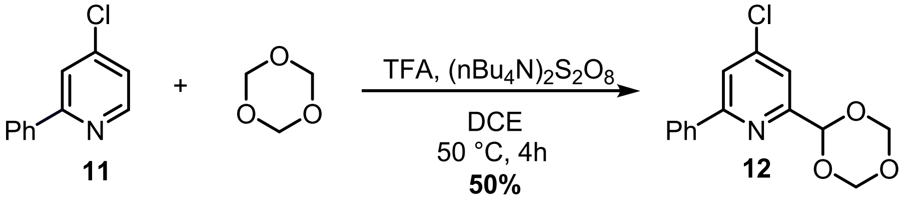

TFA (50 µL, 0.64 mmol, 1.0 equiv.), 1,3,5-trioxane (588 mg, 6.4 mmol, 10.0 equiv.) and $(nBu_4N)_2S_2O_8$ (1.512 g, 2.24 mmol, 3.4 equiv.) were subsequently added to a solution of 4-chloro-2-phenylpyridine **11** (124 mg, 0.64 mmol, 1.0 equiv.) in DCE (2.16 mL, 0.3 M). The mixture was stirred at 50 °C for 4 hours then quenched with saturated $NaHCO_3$. The phases were separated and the organic layer concentrated *in vacuo* and purified by column chromatography (hex:EtOAc 90:10) to afford compound **12** as a white solid (88 mg, 0.316 mmol, 50% yield). ^{1}H NMR (400 MHz, $CDCl_3$) δ: 7.97 (dd, J = 8.0, 1.6 Hz, 2H), 7.73 (d, J = 1.8 Hz, 1H), 7.67 (d, J = 1.8 Hz, 1H), 7.53 – 7.41 (m, 3H), 6.01 (s, 1H), 5.40 (d, J = 6.3 Hz, 2H), 5.37 (d, J = 6.3 Hz, 2H). ^{13}C NMR (101 MHz, $CDCl_3$) δ: 158.6, 156.7, 145.9, 137.9, 129.8, 129.0, 127.3, 121.6, 120.1, 101.4, 93.7. HRMS: 277.0507 [M+H$^+$], theoretical 277.0506.

See spectrum

Synthesis of 4-methoxy-2-phenyl-6-(1,3,5-trioxan-2-yl)pyridine (13) from 12

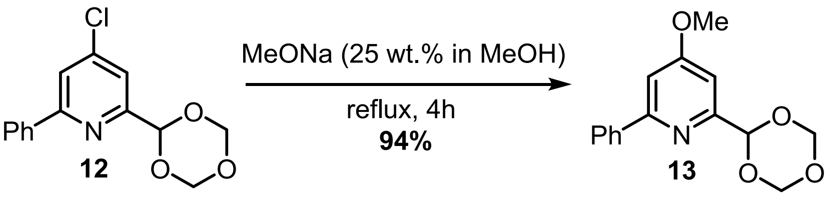

Compound **12** (67 mg, 0.24 mmol, 1.0 equiv.) was dissolved in a 25 wt. % solution of MeONa in MeOH (5.0 mL, 22.0 mmol, excess) in a round bottom flask equipped with a condenser. The reaction

was heated at reflux (65 °C) for 4 hours then concentrated *in vacuo*. A 1 M aqueous solution of $NaHSO_4$ was added, and the resulting precipitate was filtered to afford pure compound **13** as a white solid (62 mg, 0.23 mmol, 94% yield). ^{1}H NMR (400 MHz, $CDCl_3$) δ: 7.95 (dd, *J* = 8.29, 1.48 Hz, 2H), 7.50 - 7.32 (m, 3H), 7.23 (d, *J* = 2.4 Hz, 1H), 7.21 (d, *J* = 2.4 Hz, 1H), 6.00 (s, 1H), 5.40 (d, *J* = 6.3 Hz, 2H), 5.38 (d, *J* = 6.3 Hz, 2H), 3.95 (s, 3H). ^{13}C NMR (101 MHz, $CDCl_3$) δ: 167.5, 158.9, 157.2, 139.2, 129.2, 128.8, 127.3, 108.4, 104.9, 102.2, 93.8, 55.6. HRMS: 274.1071 [M+H$^+$], theoretical 274.1074. ***See spectrum***

Synthesis of 4-methoxy-2-phenylpyridine (7) from 4-methoxypyridine (14)

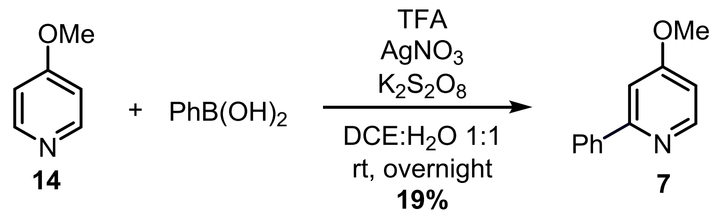

4-methoxy-pyridine **14** (20 µL, 0.23 mmol, 1.0 equiv.) was dissolved in DCE (0.88 mL, 0.27 M), and TFA (17 µL, 0.23 mmol, 1.0 equiv.) and phenylboronic acid (42 mg, 0.34 mmol, 1.5 equiv.) were subsequently added to the solution. H_2O (0.88 mL, 0.27 M) was then added, followed by $AgNO_3$ (14 mg, 0.09 mmol, 0.40 equiv.) and $K_2S_2O_8$ (185 mg, 0.69 mmol, 3.0 equiv.). The reaction was kept at room temperature overnight until completion. Then, the phases were separated and the aqueous phase was extracted with CH_2Cl_2 (2x 15 mL). The collected organic phases were dried over Na_2SO_4, filtered and concentrated *in vacuo*. The crude was purified by column chromatography (CH_2Cl_2:MeOH 95:5) to afford compound **7** as a white solid (8 mg, 0.04 mmol, 19% yield). ***See spectrum***

Synthesis of 4-methoxy-2-phenyl-6-(1,3,5-trioxan-2-yl)pyridine (13) from 7

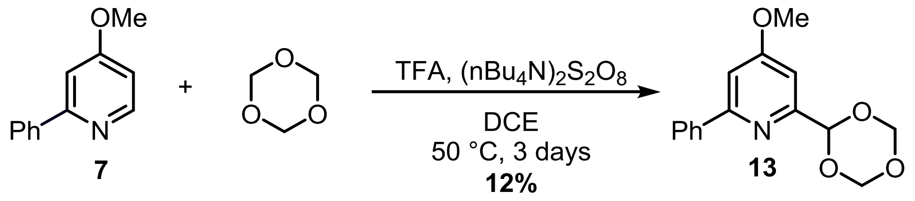

TFA (14 µL, 0.18 mmol, 1.0 equiv.), 1,3,5-trioxane (162 mg, 1.80 mmol, 10.0 equiv.) and $(nBu_4N)_2S_2O_8$ (413 mg, 0.61 mmol, 3.4 equiv.) were subsequently added to a solution of 4-methoxy-2-phenylpyridine **7** (33 mg, 0.18 mmol, 1.0 equiv.) in DCE (1.8 mL, 0.1 M). The mixture was stirred at 50 °C for 3 days and then quenched with saturated $NaHCO_3$. The phases were separated and the organic layer concentrated *in vacuo* and purified by column chromatography (toluene:EtOAc 95:15) to afford compound **13** as a white solid (6 mg, 0.021 mmol, 12% yield).

See spectrum

Synthesis of caerulomycin K from 13

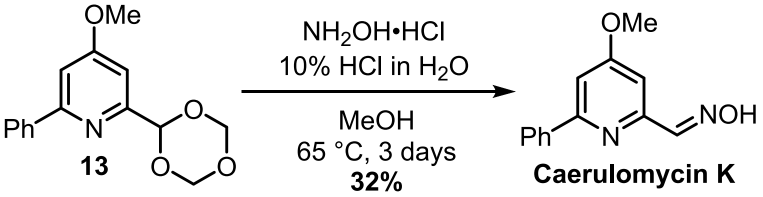

Compound **13** (60 mg, 0.22 mmol, 1.0 equiv.) was dissolved in MeOH (0.40 mL, 0.5 M), followed by the addition of $NH_2OH{\cdot}HCl$ (92 mg, 1.32 mmol, 6.0 equiv.) and of a 10% solution of HCl in H_2O (0.8 mL, 2.2 mmol, 10.0 equiv.). The reaction was stirred at 65 °C for 3 days then quenched with saturated $NaHCO_3$. The phases were separated and the organic layer concentrated *in vacuo* and purified by column chromatography (CH_2Cl_2:MeOH 97:3) to afford **caerulomycin K** as a white solid (16 mg, 0.07 mmol, 32% yield). ^1H NMR (400 MHz, DMSO) δ: 11.66 (s, 1H), 8.12 (d, J = 4.5 Hz, 1H), 8.13 – 8.06 (m, 2H), 7.53 – 7.41 (m, 4H), 7.26 (d, J = 2.2 Hz, 1H), 3.94 (s, 3H). ^{13}C NMR (101 MHz, DMSO) δ: 166.5, 157.5, 153.4, 148.9, 138.1, 129.6, 128.5, 126.7, 106.8, 103.8, 55.5. The data are consistent with those reported in the literature.[9]

See spectrum

Synthesis of caerulomycin K from 12

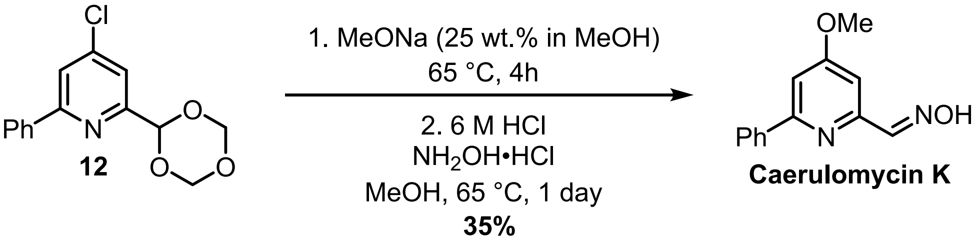

Compound **12** (80 mg, 0.29 mmol, 1.0 equiv.) was dissolved in a 25 wt. % solution of MeONa in MeOH (5.0 mL, 22.0 mmol, excess) in a round bottom flask equipped with a condenser. The reaction was heated at reflux (65 °C) for 4 hours (full conversion as monitored by TLC). The reaction mixture was cooled down to 0 °C, followed by the addition of 6 M HCl (5 mL). Then, $NH_2OH{\cdot}HCl$ (200 mg, 2.9 mmol, 10.0 equiv.) was added and the reaction was stirred at 65 °C for 1 day. The reaction mixture was then cooled down to 0 °C, followed by neutralization with 6 M NaOH. Then, the solvent was removed under reduced pressure and the crude dissolved in a CH_2Cl_2:H_2O 1:1 mixture. The two phases were separated and the organic layer was dried over Na_2SO_4, filtered, concentrated and purified by column chromatography (CH_2Cl_2:MeOH 97:3) to afford **caerulomycin K** as a white solid (23 mg, 0.010 mmol, 35% yield).

See spectrum

3. SPECTROSCOPIC DATA

^{1}H NMR (400 MHz, $CDCl_3$) of **2** (*see procedure*)

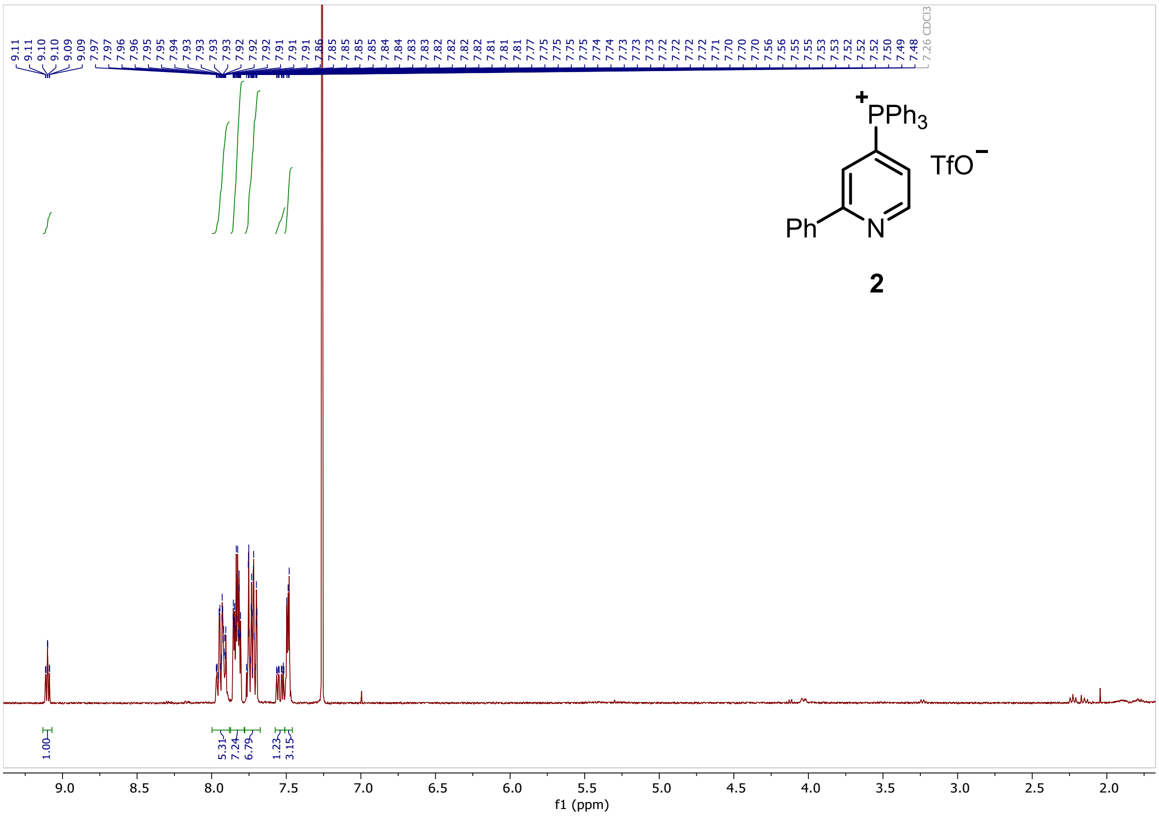

^{31}P NMR (162 MHz, $CDCl_3$) of **2**

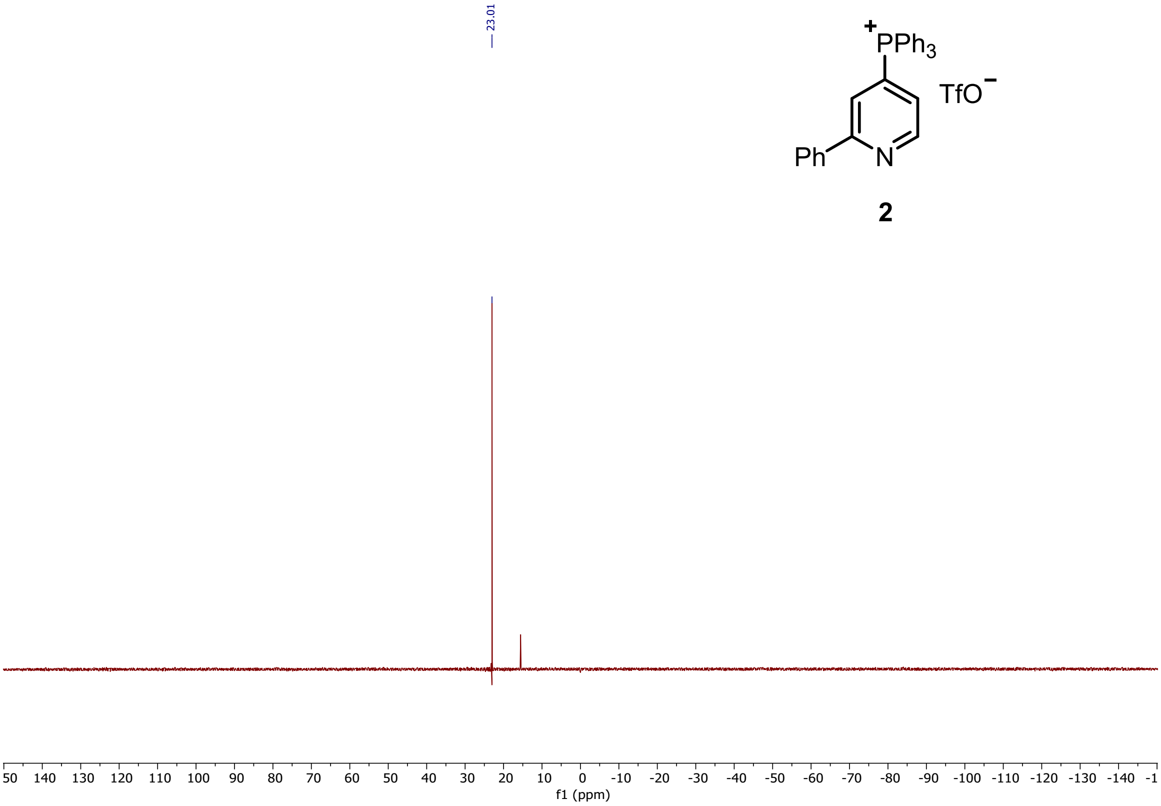

^{1}H NMR (400 MHz, $CDCl_3$) of **5** (*see procedure*)

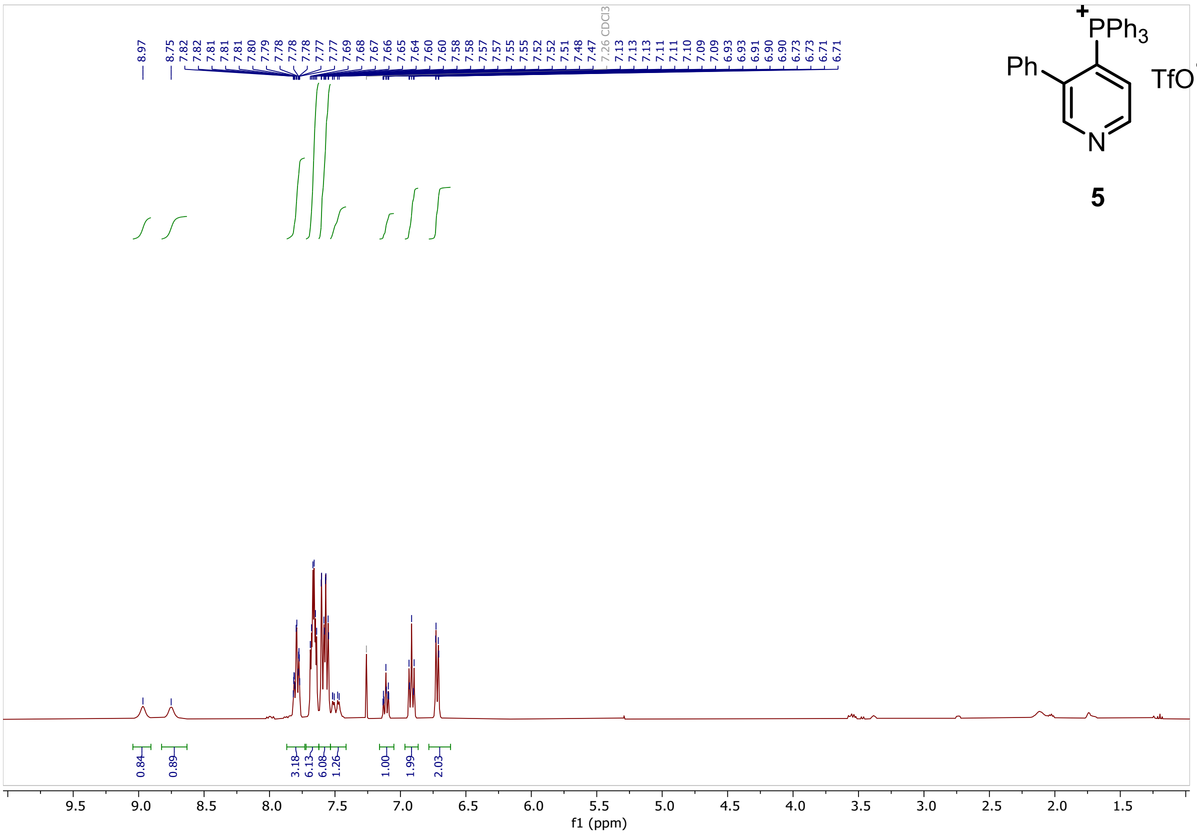

^{31}P NMR (162 MHz, $CDCl_3$) of **5**

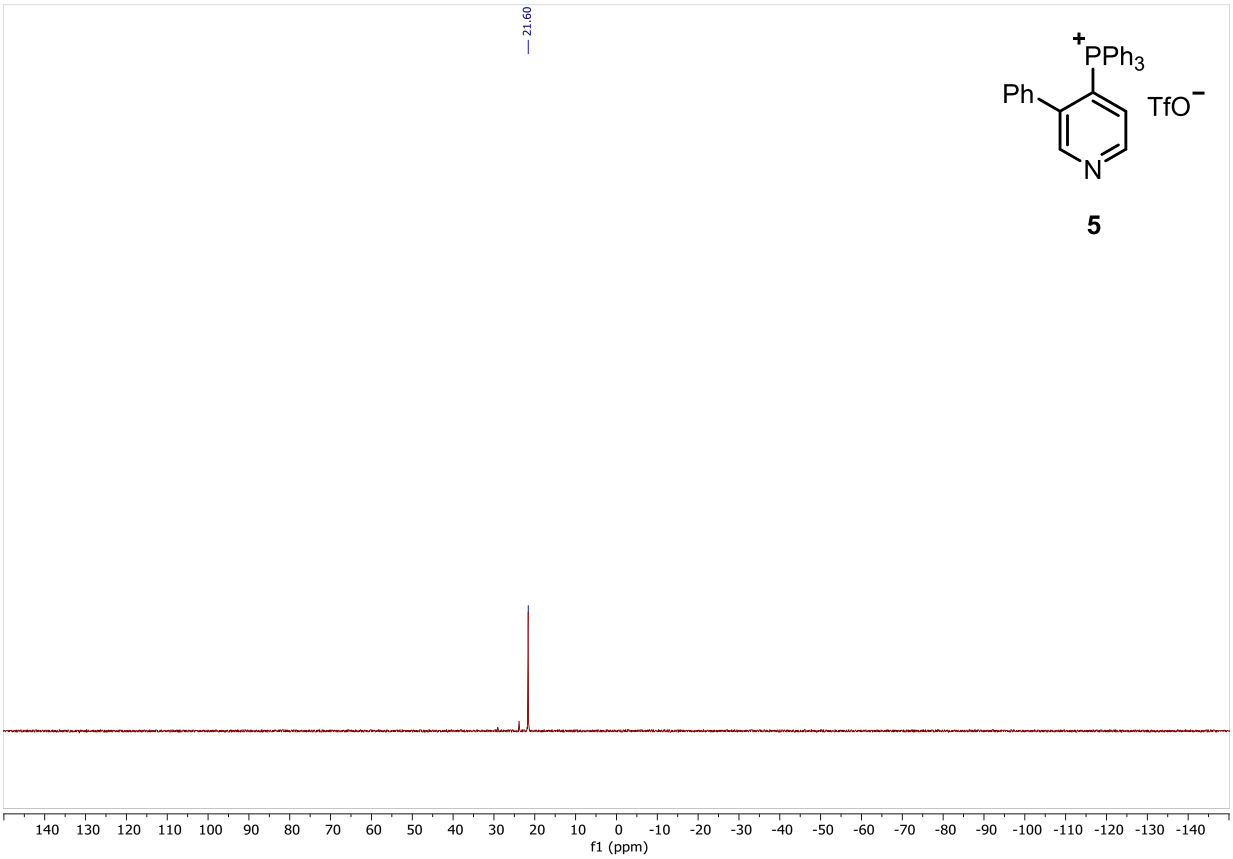

^{1}H NMR (400 MHz, $CDCl_3$) of **7** (*see procedure*)

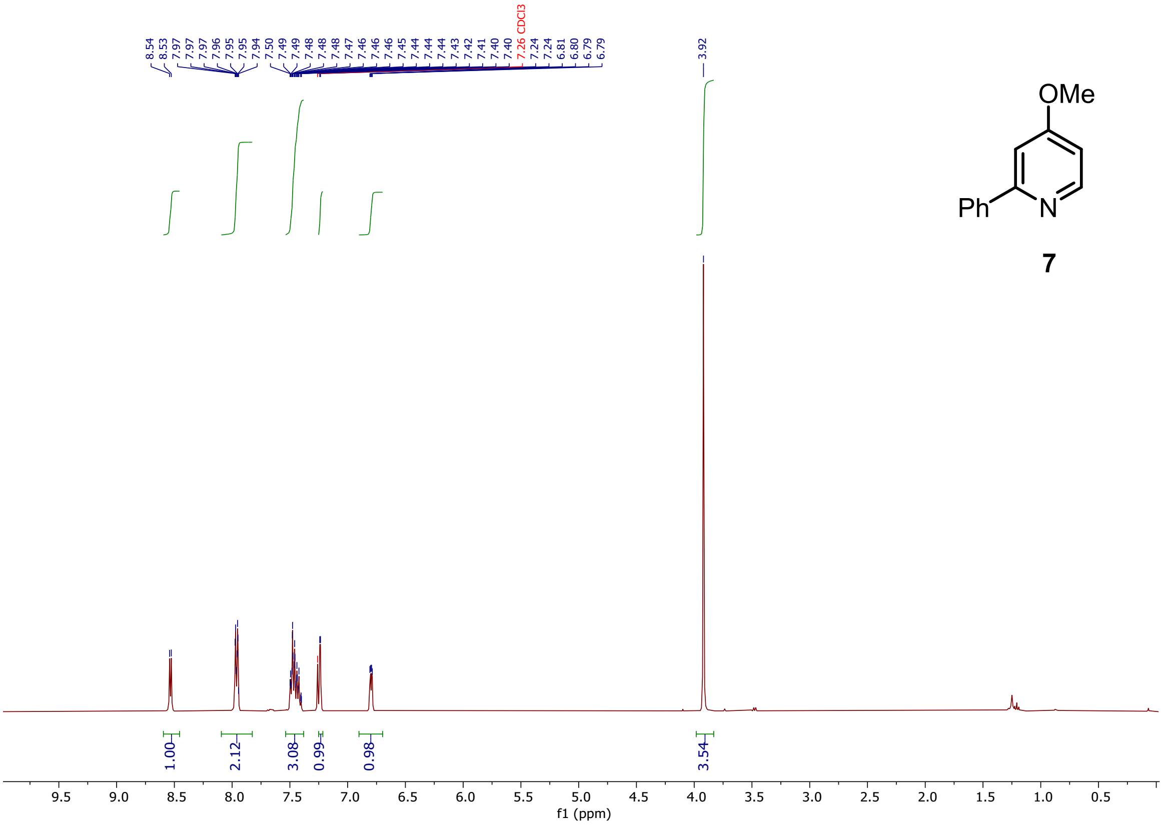

^{1}H NMR (400 MHz, $CDCl_3$) of **1-O** (*see procedure*)

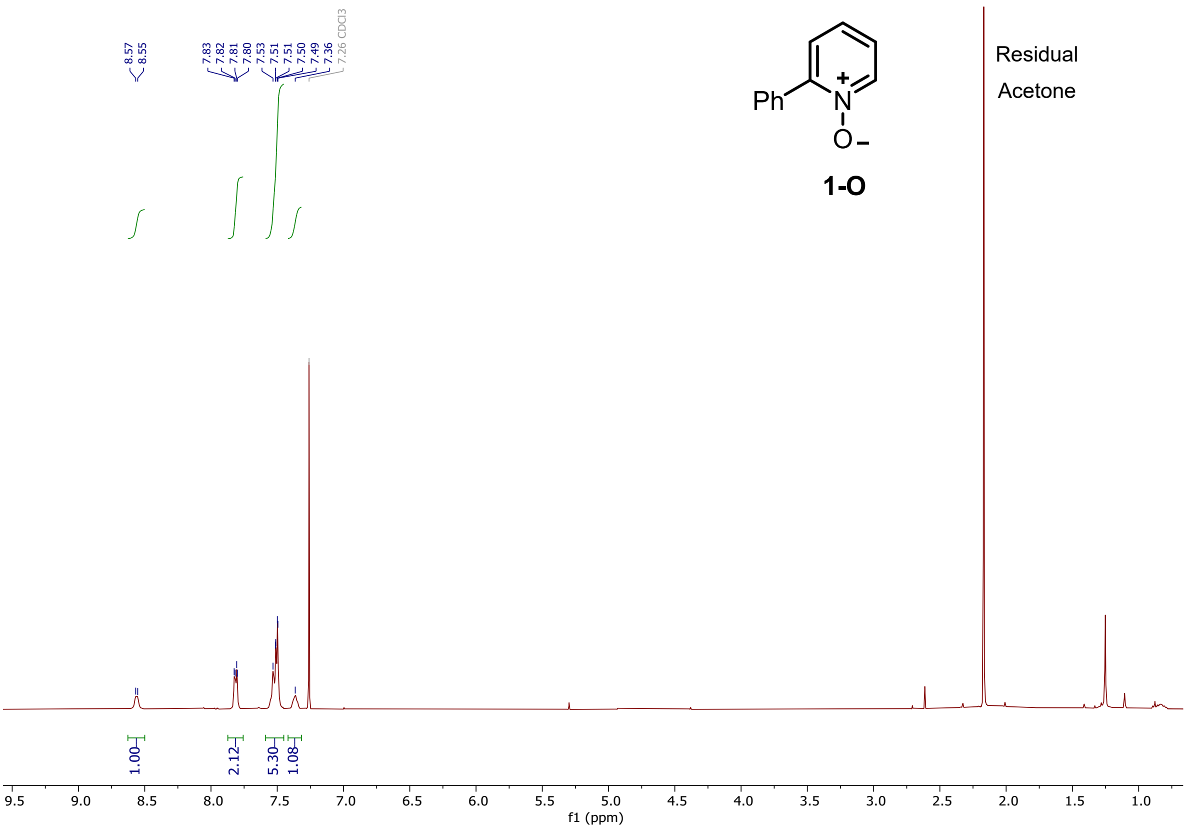

^{1}H NMR (400 MHz, $CDCl_3$) of 5-bromo-2-phenylpyridine (*see procedure*)

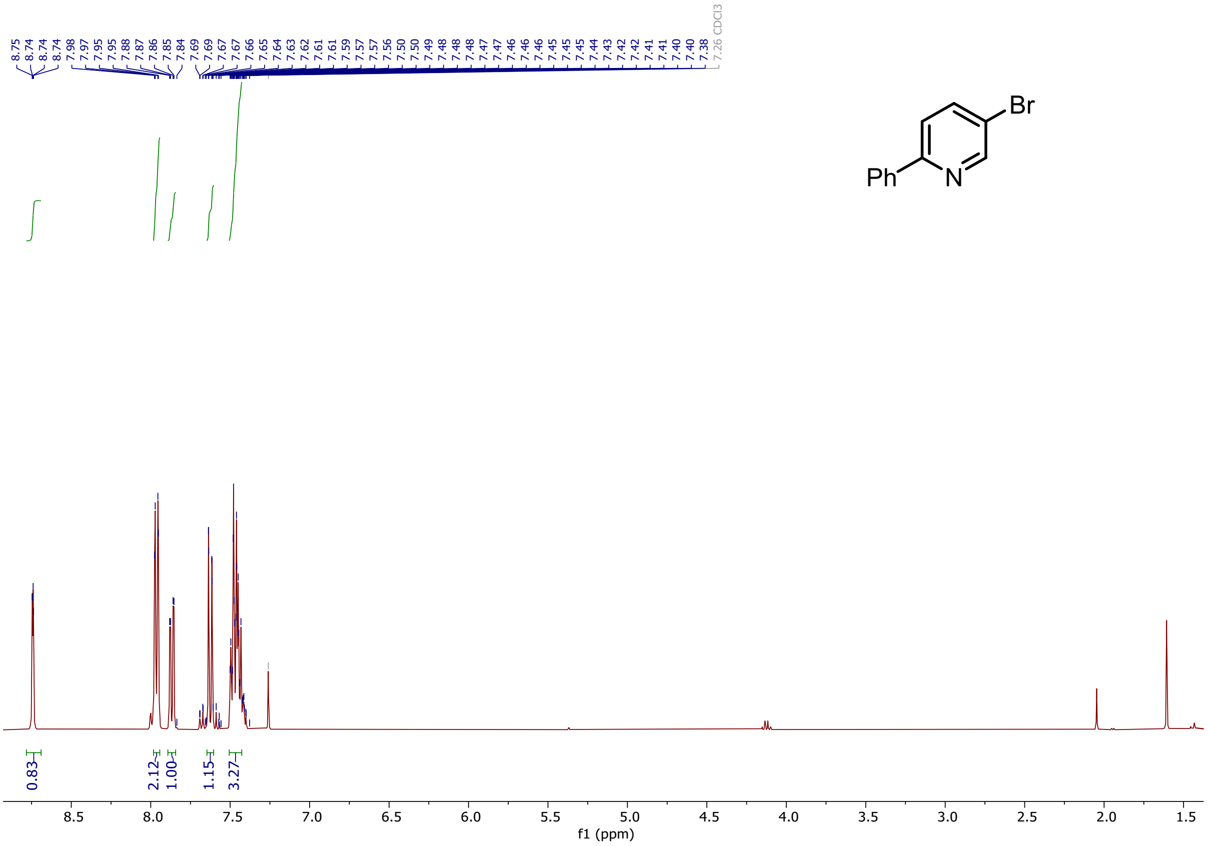

^{1}H NMR (400 MHz, $CDCl_3$) of 4-bromo-2-phenylpyridine (*see procedure*)

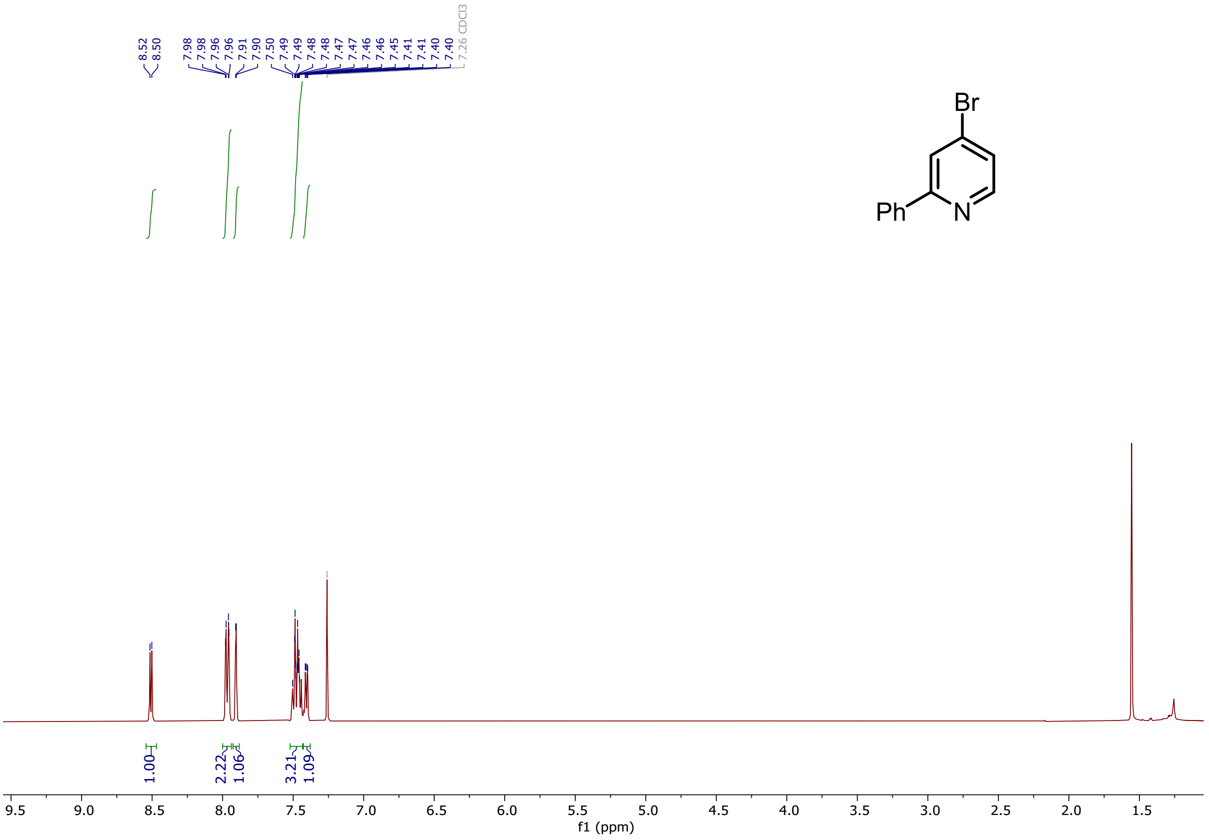

^{1}H NMR (400 MHz, $CDCl_3$) of 3-bromo-2-phenylpyridine (*see procedure*)

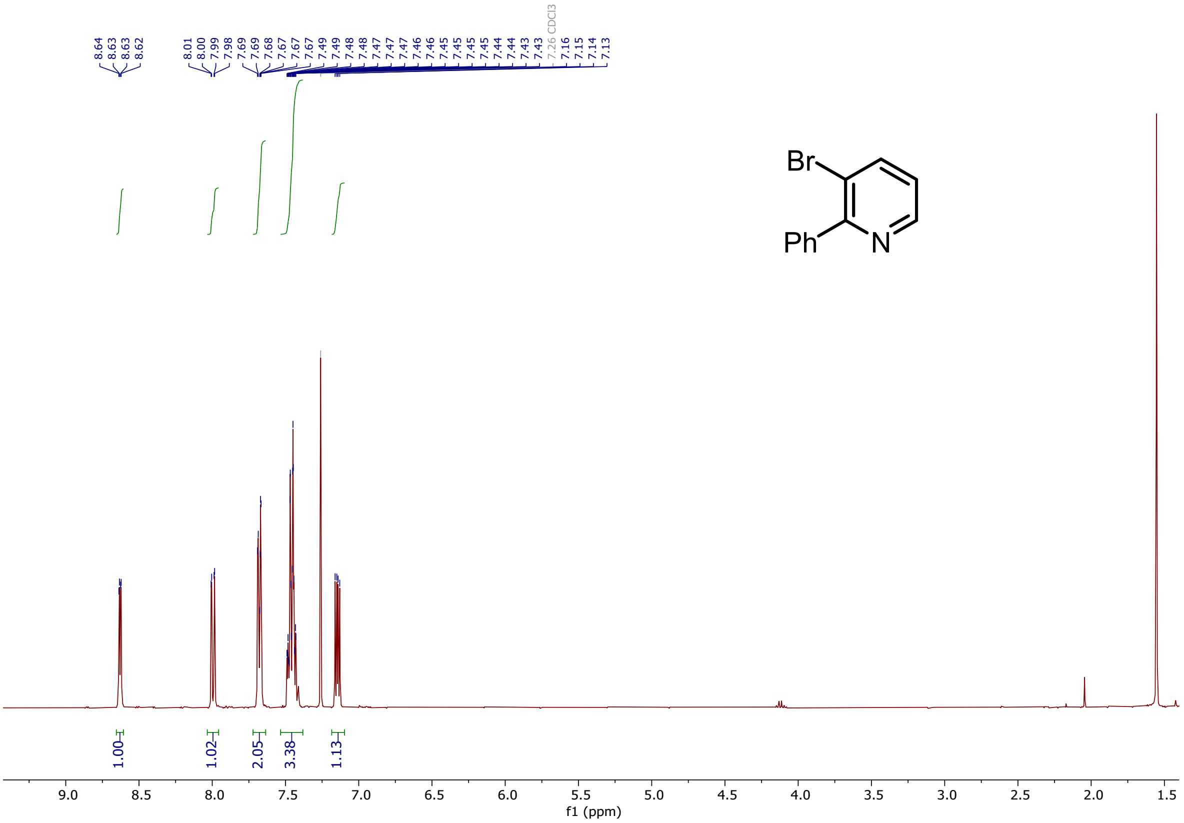

^{1}H NMR (400 MHz, $CDCl_3$) of **9** (*see procedure*)

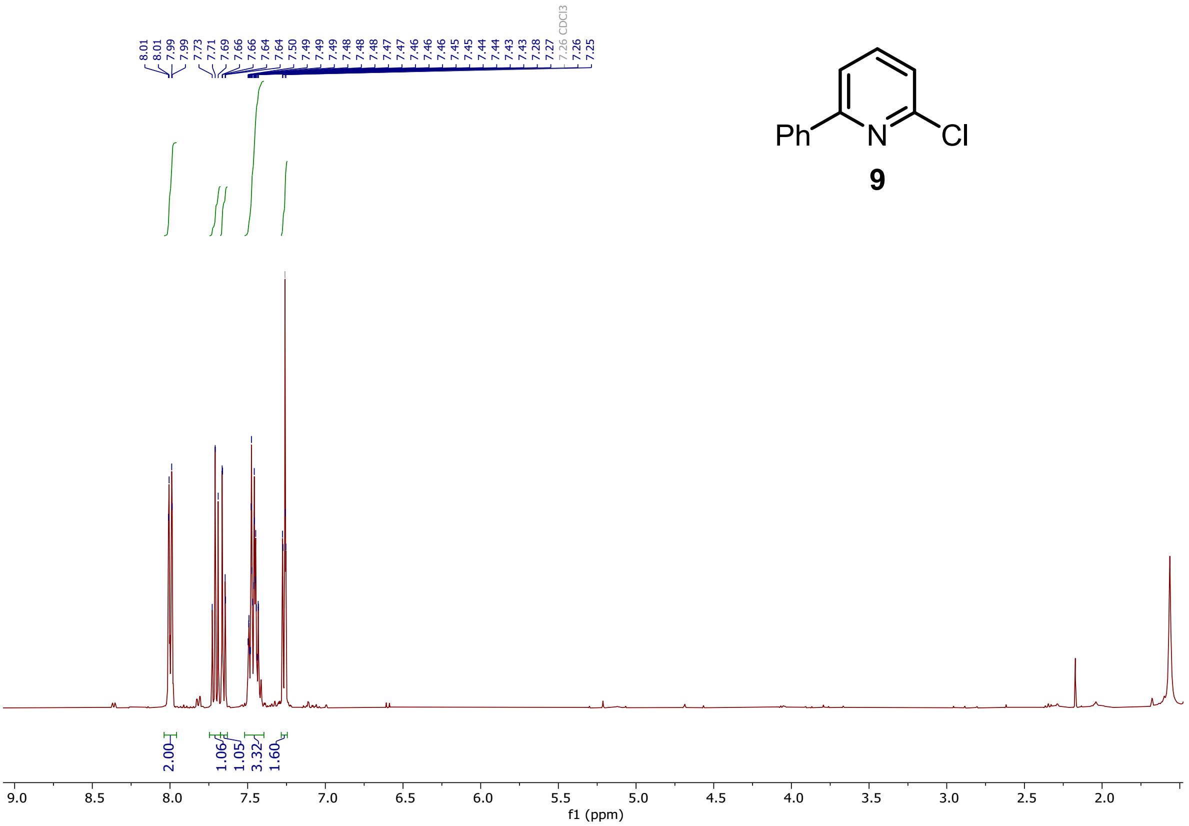

^{1}H NMR (400 MHz, $CDCl_3$) of **11** (*see procedure*)

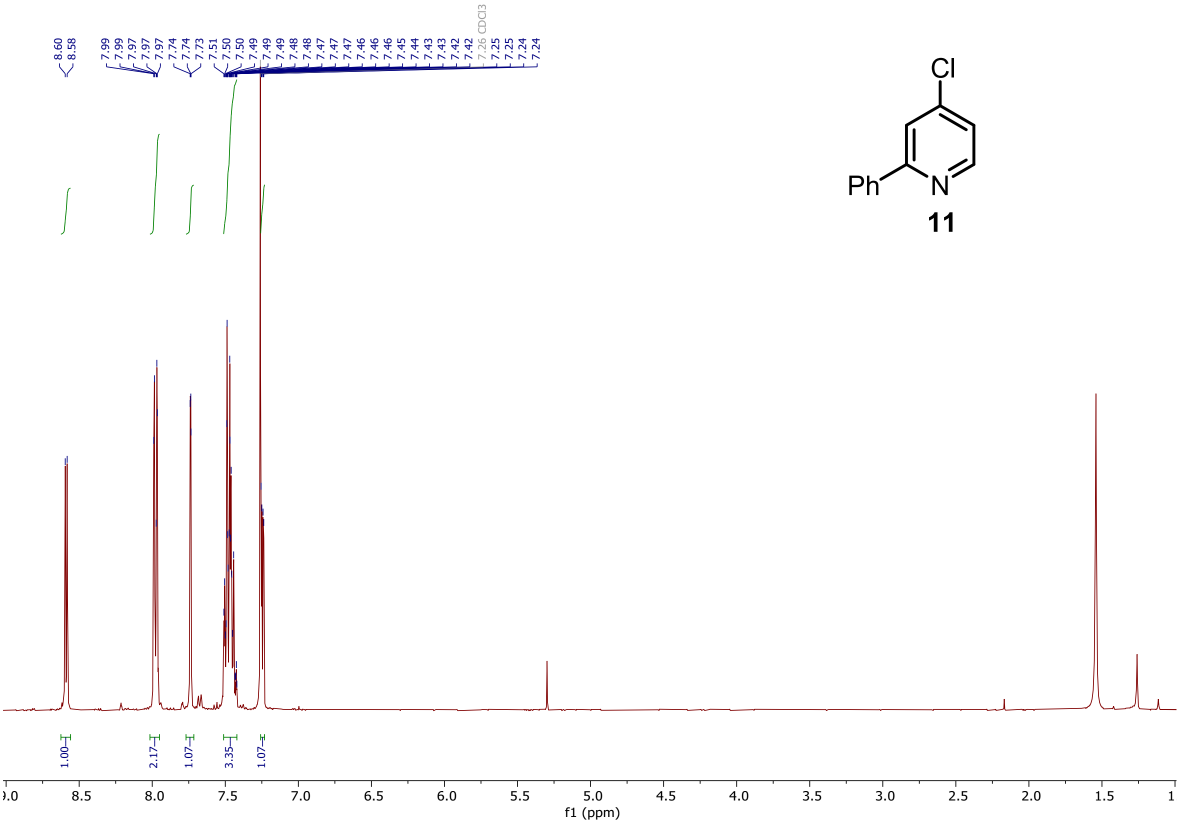

^{1}H NMR (400 MHz, $CDCl_3$) of **12** (*see procedure*)

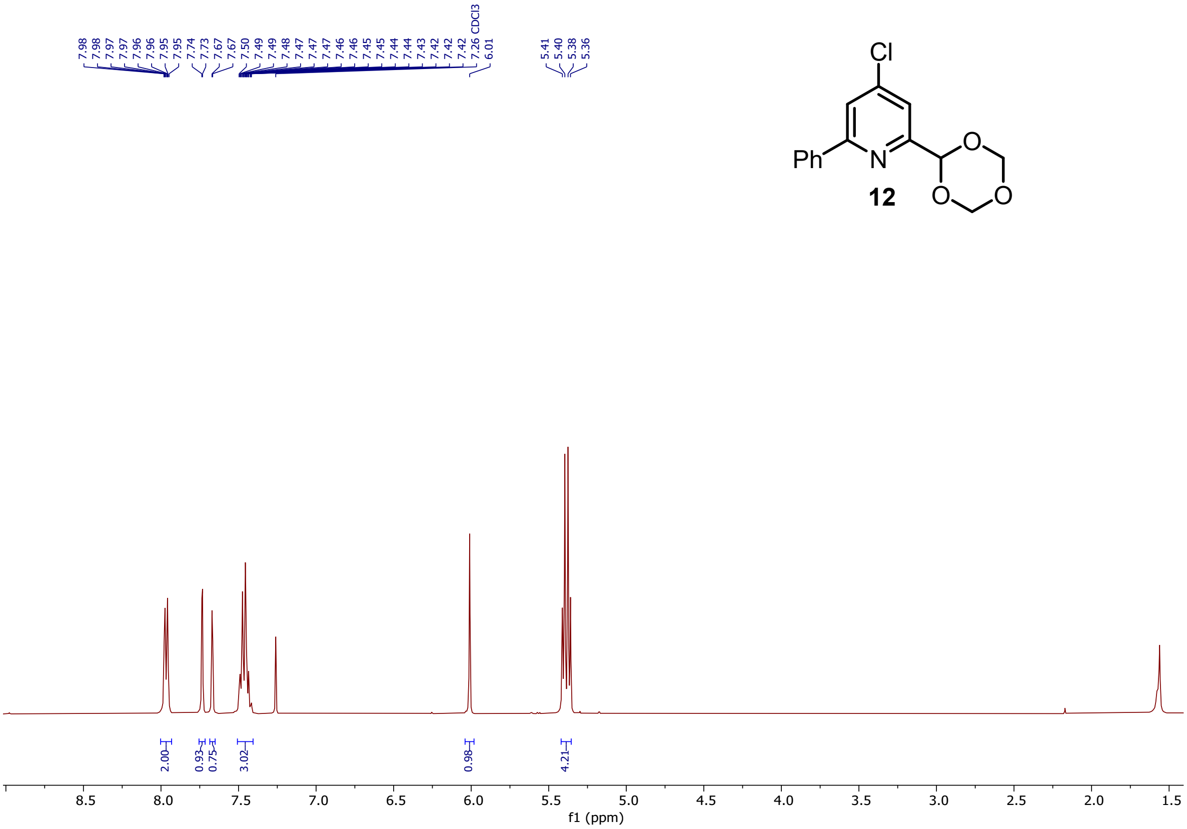

^{13}C NMR (101 MHz, $CDCl_3$) of **12**

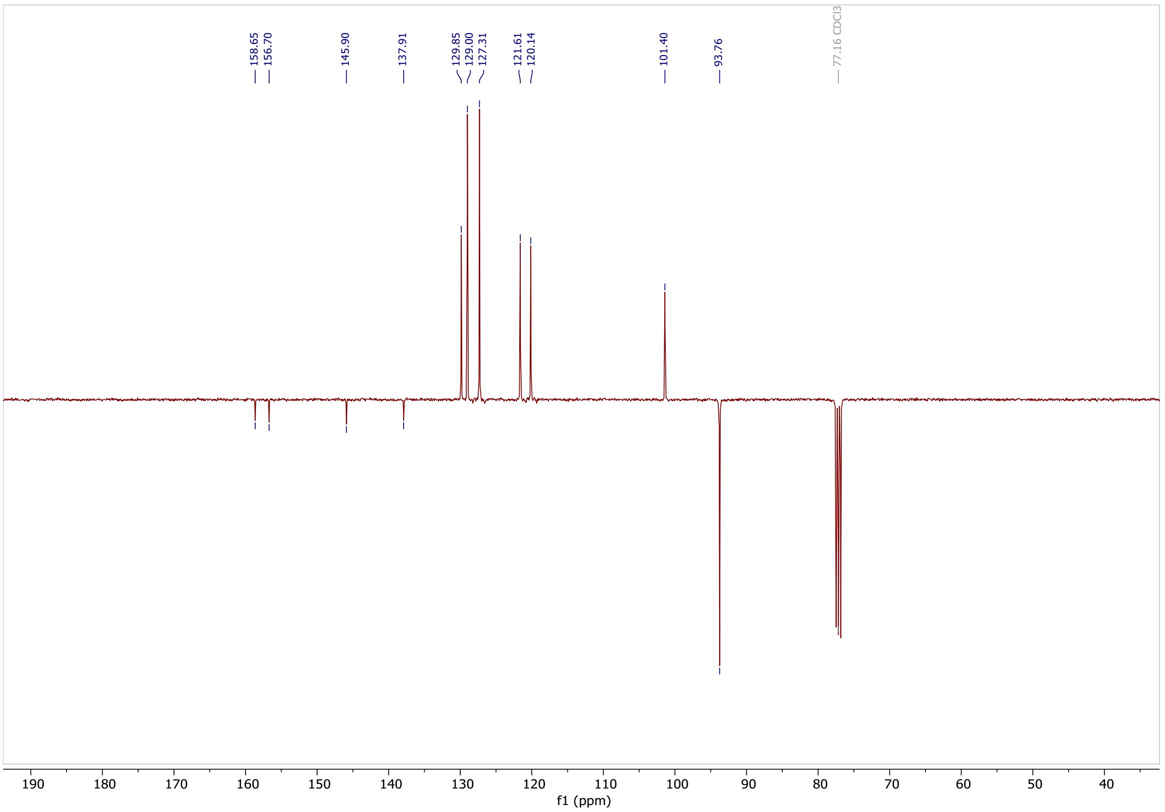

^{1}H NMR (400 MHz, $CDCl_3$) of **13** (*see procedure*)

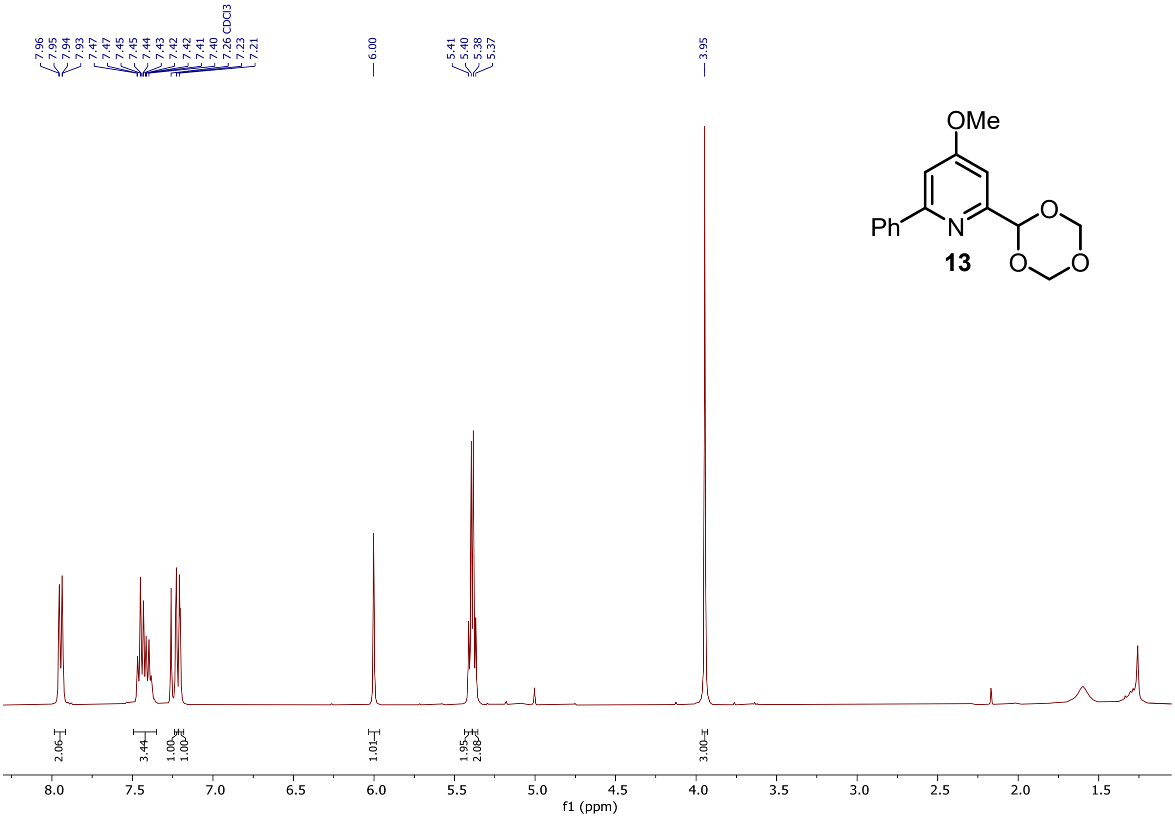

^{13}C NMR (101 MHz, $CDCl_3$) of **13**

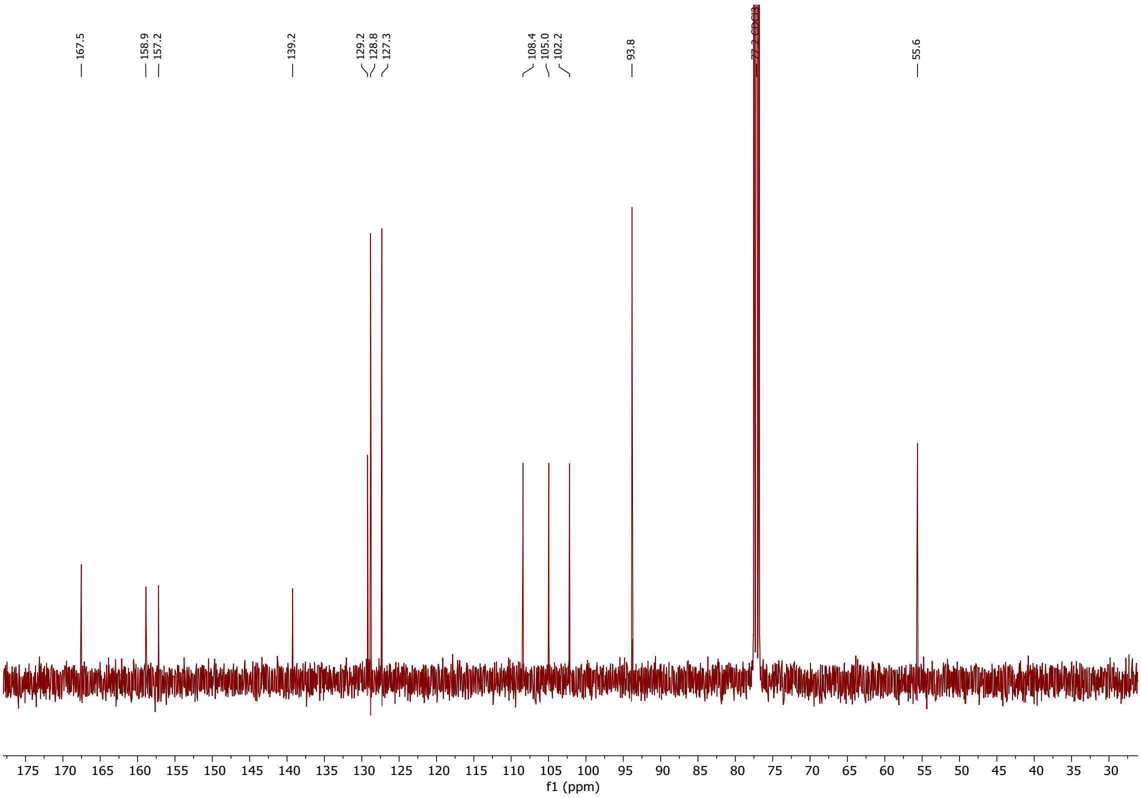

COSY (in $CDCl_3$) of **13**

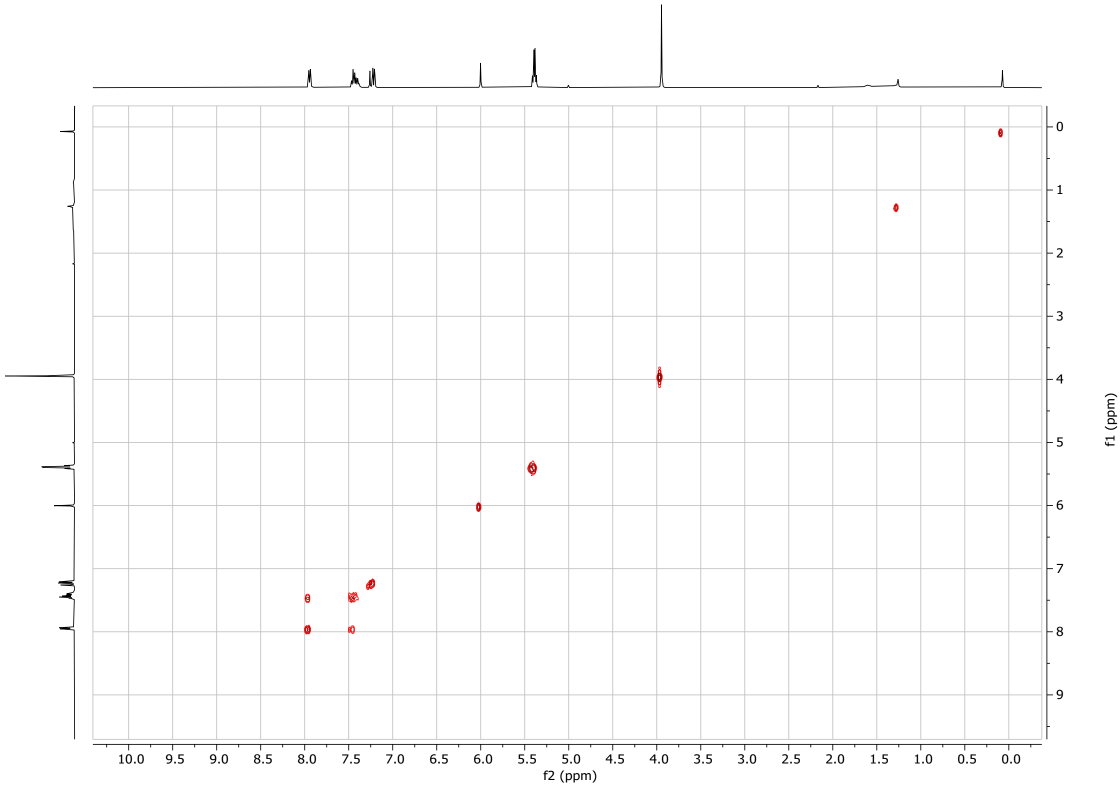

HSQC (in $CDCl_3$) of **13**

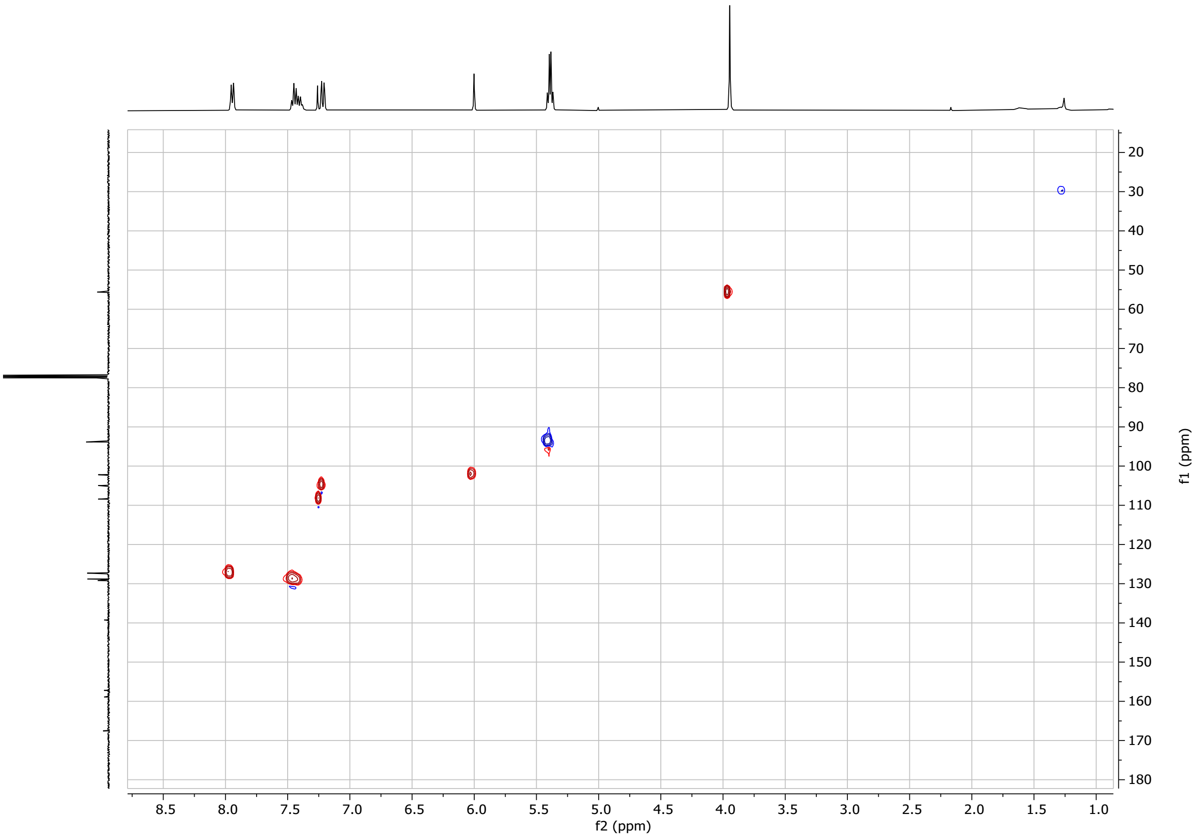

HMBC (in $CDCl_3$) of **13**

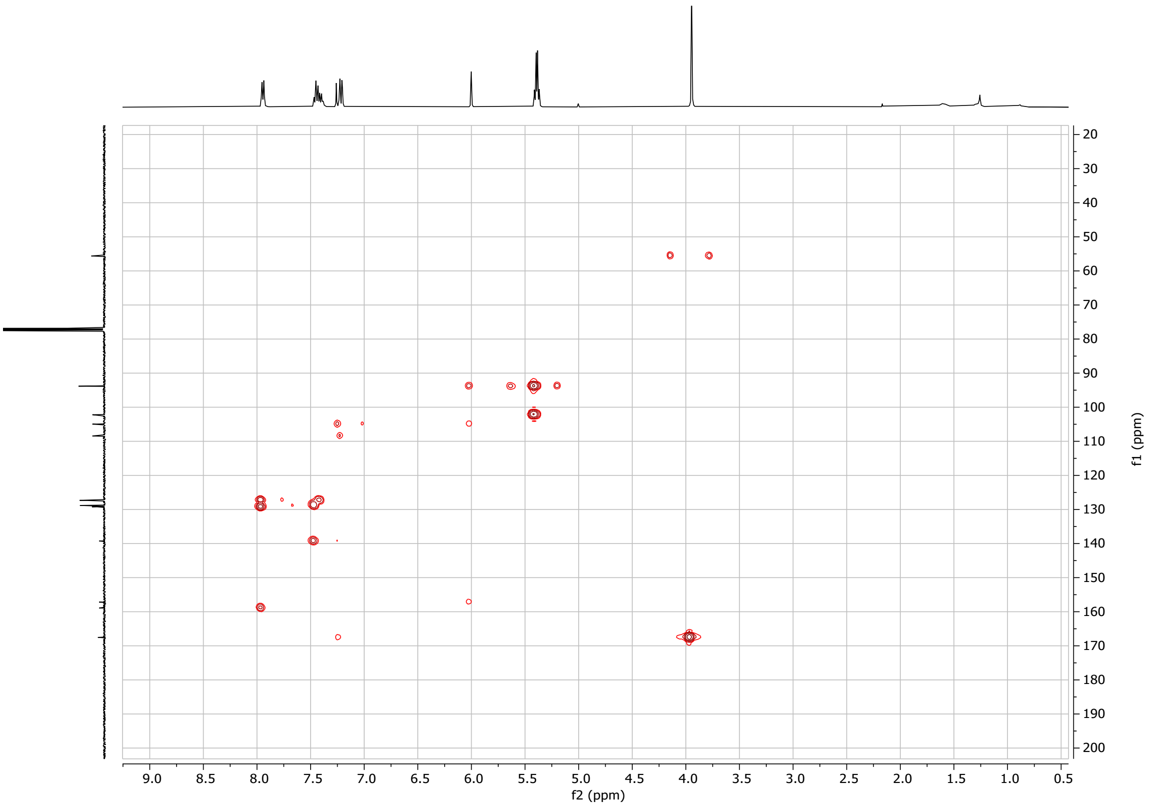

^{1}H NMR (400 MHz, DMSO) of **caerulomycin K** (*see procedure*)

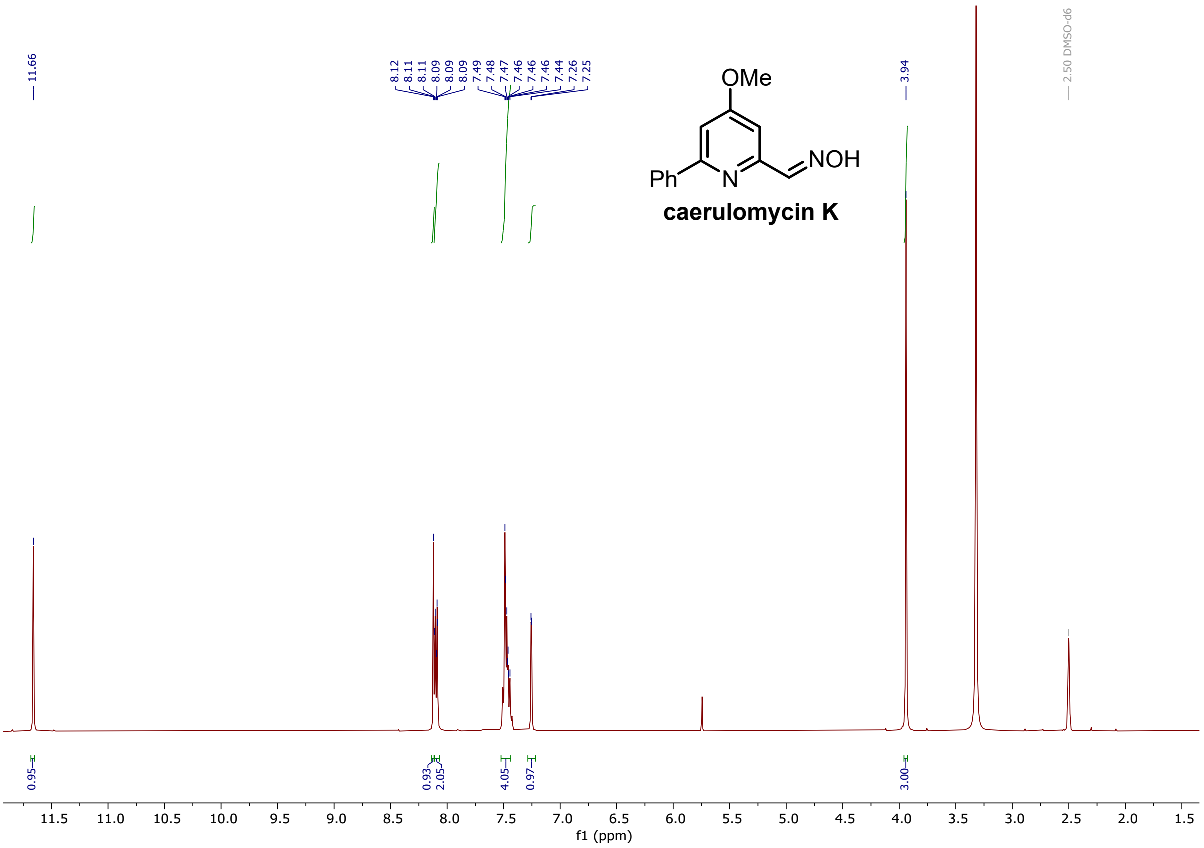

^{13}C NMR (101 MHz, DMSO) of **caerulomycin K**

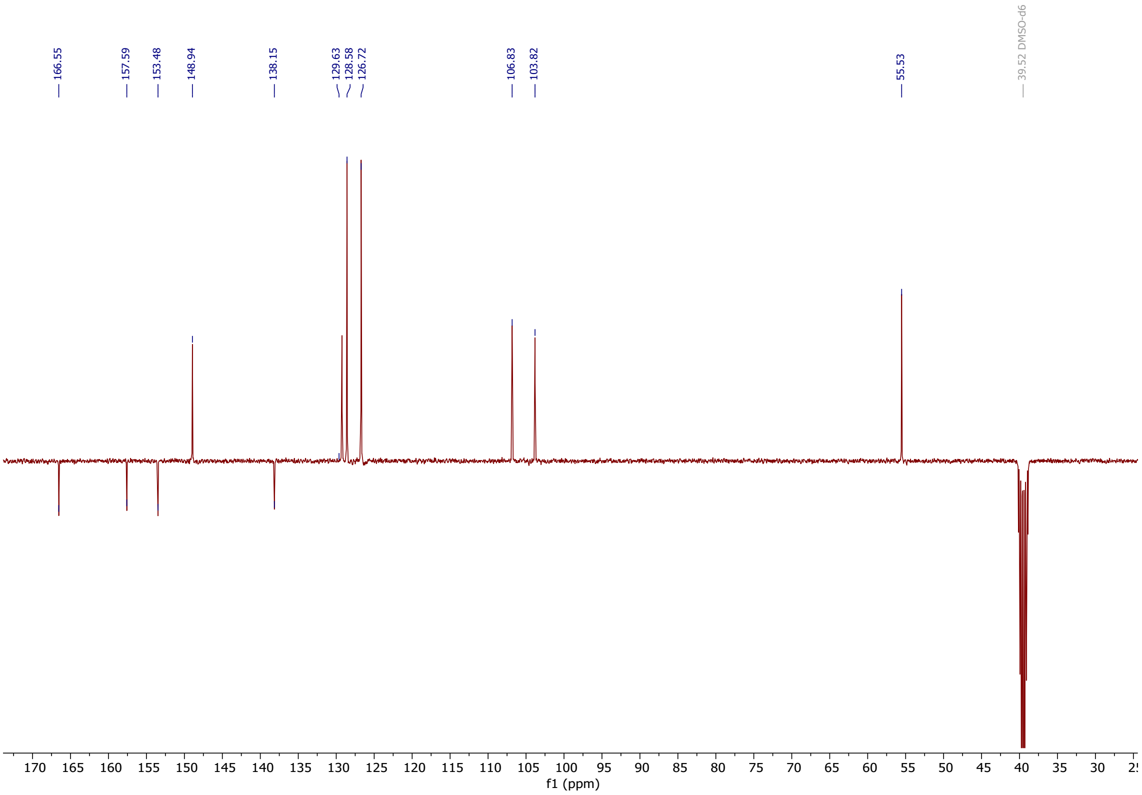

4. REFERENCES

[1] M. C. Hilton, R. D. Dolewski, A. McNally, *J. Am. Chem. Soc.* **2016**, *138,* 13806-13809.

[2] K. Xu, F. Yang, G. Zhang, Y. Wu, *Green Chem.* **2013**, *15,* 1055-1060.

[3] W. S. Tay, Y. Li, X.-Y. Yang, S. A. Pullarkat, P.-H. Leung, *J. Organomet. Chem*. **2020**, *914*, 121216.

[4] A. J. Paterson, C. J. Hron, C. L. McMullin, M. F. Mahon, N. J. Press, C. G. Frost, *Org. Biomol. Chem*. **2017**, *15,* 5993-6000.

[5] Y. X. Chen, J.-T. He, N.-C. Wu, Z.-L. Liu, P.-J. Xia, K. Chen, H.-J. Xiang, H. Yang, *Chem. Commun.* **2023**, *59*, 6588-6591.

[6] M. Dabiri, S. I. Alavioon, S. K. Movahed, *Eur. J. Org. Chem*. **2019**, *2019*, 1479-1487.

[7] Q. Zhou, B. Zhang, L. Su, T. Jiang, R. Chen, T. Du, Y. Ye, J. Shen, G. Dai, D. Han, H. Jiang, *Tetrahedron* **2013**, *69*, 10996-11003.

[8] L. Hintermann, T. T. Dang, A. Labonne, T. Kribber, L. Xiao, P. Naumov, *Chem. Eur. J*. **2009**, *15*, 7167-7179.

[9] P. Fu, S. Wang, K. Hong, X. Li, P. Liu, Y. Wang, W. Zhu, *J. Nat. Prod.* **2011**, *74*, 1751-1756.
